# Correction: Accurate and Robust Genomic Prediction of Celiac Disease Using Statistical Learning

**DOI:** 10.1371/journal.pgen.1004374

**Published:** 2014-04-14

**Authors:** The PLOS Genetics Staff

## Notice of Republication

This article was republished on March 24, 2014 to correct an error in the formatting of Figure 4. Please download this article again to view the correct version. The originally published, uncorrected article and the republished, corrected article are provided here for reference.

## Supporting Information

File S1 Originally published, uncorrected article.

File S2 Republished corrected article.

## References

[1] Abraham G, Tye-Din JA, Bhalala OG, Kowalczyk A, Zobel J, et al (2014) Accurate and Robust Genomic Prediction of Celiac Disease Using Statistical Learning. PLoS Genet 10(2): e1004137 doi:10.1371/journal.pgen.1004137 24550740 10.1371/journal.pgen.1004137 PMC3923679

