# Supplementary material for: Accurate and Robust Genomic Prediction of Celiac Disease Using Statistical Learning
Source: PLoS Genet. 2014 Feb 13;10(2):e1004137. doi: 10.1371/journal.pgen.1004137 (PMC3923679; doi:10.1371/journal.pgen.1004137)
Supplement: Table S1 — The predictive model. The SNPs are sorted in decreasing order of the absolute value of their model weight averaged over the 10×10 cross-validation folds. Stability is the percentage of times a SNP was selected to have non-zero weight over the 10×10-cross-validation folds. Intercept: −0.757226. To annotate the SNPs we used Bioconductor 2.12 together with the packages VariantAnnotation 1.6.5 and TxDb.Hsapiens.UCSC.hg18.knownGene 2.9.0. We considered a SNP to be genic if it was annotated to fall inside one of the regions {spliceSite, intron, fiveUTR, threeUTR, coding, promoter} and intergenic otherwise. For intergenic SNPs, we also annotate the nearest gene and the distance to it. All positions are in hg18 coordinates. (PDF) [file pgen.1004137.s006.pdf]

Supplementary Table 1

#	RS	Chr	BP	Ref	Weight	Stab. (%)	GeneSymbol	Location	Dist. (bp)
1	rs2187668	6	32713862	T	0.213782	100	HLA-DQA1	Genic	
2	rs9357152	6	32772938	C	−0.151141	100	HLA-DQB1	Intergenic	30575
3	rs3099844	6	31556955	T	0.095107	100	MICB	Intergenic	16994
4	rs3129962	6	32487361	A	0.091648	88	BTNL2	Intergenic	4482
5	rs3129763	6	32698903	T	0.088432	100	HLA-DQA1	Intergenic	14310
6	rs1063355	6	32735692	A	−0.086255	100	HLA-DQB1	Genic	
7	rs2858308	6	32777978	T	0.057213	100	HLA-DQB1	Intergenic	35615
8	rs7765379	6	32788906	C	−0.048124	100	HLA-DQA2	Intergenic	28292
9	rs1794282	6	32774504	T	0.047955	100	HLA-DQB1	Intergenic	32141
10	rs9275224	6	32767856	C	−0.047468	100	HLA-DQB1	Intergenic	25493
11	rs2064478	6	33180244	T	0.043888	98	HLA-DPB2	Intergenic	8029
12	rs204999	6	32217957	C	0.037645	100	PRRT1	Intergenic	6510
13	rs17211510	6	32710408	A	−0.035793	100	HLA-DQA1	Intergenic	2805
14	rs9368699	6	31910520	G	−0.035638	89	C6orf48, SNORD48	Genic	
15	rs2856997	6	32889754	T	−0.026079	100	HLA-DOB	Genic	
16	rs17620389	6	38642407	G	0.025118	70	BTBD9	Genic	
17	rs13098911	3	46210205	T	0.024216	96	CCR3	Genic	
18	rs1559810	3	189607048	T	0.023239	99	LPP	Genic	
19	rs9851967	3	189570322	T	−0.023087	100	LPP	Genic	
20	rs2327832	6	138014761	C	0.022413	99	OLIG3	Intergenic	157760
21	rs429916	6	33086565	A	0.020479	99	HLA-DOA	Genic	
22	rs2162610	2	204426974	C	0.020260	97	CTLA4	Intergenic	13936
23	rs6090314	20	61327997	T	0.019180	96	BIRC7	Intergenic	9896
24	rs10798176	1	170942148	C	−0.017969	97	FASLG	Intergenic	40368
25	rs302483	5	88179847	G	−0.017305	57	MEF2C	Genic	
26	rs13100170	3	97139125	G	0.017101	90	EPHA6	Intergenic	877032
27	rs2635299	4	118762806	C	−0.017007	99	NDST3	Intergenic	431707
28	rs17095255	10	118543853	A	−0.016682	61	HSPA12A	Genic	
29	rs17021444	1	104695375	C	−0.015603	90	AMY1A	Intergenic	592571
30	rs9276502	6	32827644	C	0.015514	74	HLA-DQB2	Intergenic	4563
31	rs10203407	2	66552300	T	−0.014764	33	MEIS1	Genic	
32	rs204990	6	32269408	A	0.014194	96	GPSM3	Genic	
33	rs12086216	1	104601817	T	−0.014020	69	AMY1A	Intergenic	499013
34	rs2849015	6	32306914	A	0.013608	98	NOTCH4	Intergenic	7230
35	rs16889229	4	13417061	T	0.013509	69	BOD1L1	Intergenic	178751
36	rs36253	19	8230893	G	0.013407	76	CERS4	Genic	
37	rs404890	6	32306845	A	0.013205	38	NOTCH4	Intergenic	7161
38	rs9811792	3	161179692	C	0.013039	91	IL12A	Intergenic	9845
39	rs12915188	15	98730436	A	−0.012683	88	CERS3	Intergenic	30004
40	rs1368910	2	80083549	C	−0.012608	65	CTNNA2	Genic	

(continued)

#	RS	Chr	BP	Ref	Weight	Stab. (%)	GeneSymbol	Location	Dist. (bp)
41	rs224612	11	32019805	A	−0.012604	81	RCN1	Intergenic	49513
42	rs7762279	6	32863268	C	0.012532	74	HLA-DQB2	Intergenic	24042
43	rs2031341	10	10626295	A	−0.012285	87	CELF2	Intergenic	461061
44	rs2473520	6	139472753	T	−0.011833	79	HECA	Intergenic	25473
45	rs877345	10	8093782	C	0.011817	44	TAF3	Genic	
46	rs760883	6	16733599	T	−0.011505	73	ATXN1	Genic	
47	rs6865418	5	150567539	A	−0.011444	50	CCDC69	Genic	
48	rs1889548	1	94275785	T	−0.011227	79	ABCA4	Genic	
49	rs4835459	4	148966411	G	−0.011079	12	ARHGAP10	Genic	
50	rs9276472	6	32825202	G	0.011054	66	HLA-DQA2	Intergenic	3052
51	rs1336981	6	152124062	C	0.010885	67	ESR1	Genic	
52	rs11659563	18	71979269	C	0.010824	70	ZNF516	Intergenic	224171
53	rs2503661	6	92817083	A	−0.010798	79	EPHA7	Intergenic	1192781
54	rs7734102	5	179086403	T	0.010554	75	CANX	Genic	
55	rs765324	11	10691902	A	0.010531	70	MRVI1	Intergenic	61556
56	rs8118581	20	59323981	A	0.010517	33	CDH4	Genic	
57	rs13081814	3	97031445	G	0.010094	66	EPHA6	Intergenic	984712
58	rs12455576	18	52063003	A	−0.010086	19	TXNL1	Intergenic	358252
59	rs10736156	10	104009437	G	0.009961	53	GBF1	Genic	
60	rs747860	14	64309647	A	0.009861	60	SPTB	Genic	
61	rs2459444	10	37423602	A	0.009812	79	ANKRD30A	Intergenic	31287
62	rs9378200	6	31680906	C	−0.009691	71	AIF1	Intergenic	10179
63	rs1353111	11	128780953	C	0.009623	50	BARX2	Genic	
64	rs10520062	2	11067829	T	0.009419	37	KCNF1	Intergenic	96340
65	rs11221332	11	127886184	A	0.009196	71	ETS1	Genic	
66	rs4741986	9	4413007	G	0.009190	51	SLC1A1	Intergenic	67672
67	rs17144110	7	21246477	A	−0.008975	33	SP4	Intergenic	187917
68	rs6933404	6	138000928	C	0.008966	28	OLIG3	Intergenic	143927
69	rs2442749	6	31460019	C	0.008908	69	DDX39B	Intergenic	146170
70	rs12653303	5	146863798	C	0.008758	48	DPYSL3	Genic	
71	rs7067538	10	109596730	C	0.008358	56	SORCS1	Intergenic	682455
72	rs7026544	9	129818335	T	0.008127	19	FAM102A	Intergenic	36097
73	rs3903036	11	24622561	T	−0.008114	48	LUZP2	Genic	
74	rs1367273	2	7483477	A	−0.008067	33	RNF144A	Intergenic	386134
75	rs12584486	13	42282533	C	0.007864	43	FAM216B	Intergenic	21606
76	rs12542756	8	3114526	C	0.007676	55	CSMD1	Genic	
77	rs905426	15	29870041	T	0.007668	55	OTUD7A	Genic	
78	rs2744718	1	22396314	A	0.007535	35	WNT4	Intergenic	54311
79	rs570854	6	52320209	G	0.007503	62	PAQR8	Intergenic	55761
80	rs17775775	10	115660803	G	−0.007434	21	NHLRC2	Intergenic	2517

(continued)

#	RS	Chr	BP	Ref	Weight	Stab. (%)	GeneSymbol	Location	Dist. (bp)
81	rs1596161	15	99086254	C	0.007290	35	ASB7	Intergenic	80063
82	rs2377127	5	118288687	G	0.007276	36	DTWD2	Genic	
83	rs354751	20	58338889	G	0.007180	49	C20orf197	Intergenic	259530
84	rs2301226	6	33142574	A	−0.007174	56	HLA-DPA1	Genic	
85	rs4290865	4	92883724	A	−0.007153	54	CCSER1	Intergenic	144492
86	rs13435654	4	7490276	A	0.007103	41	SORCS2	Genic	
87	rs1738074	6	159385965	T	0.007066	60	TAGAP	Genic	
88	rs6889648	5	169564839	A	0.007043	28	C5orf58	Intergenic	28878
89	rs618019	20	47840534	T	0.007005	30	SLC9A8	Intergenic	22332
90	rs10026953	4	147683155	T	0.006977	34	SLC10A7	Intergenic	20835
91	rs11203203	21	42709255	T	0.006974	57	UBASH3A	Genic	
92	rs3852253	7	18832715	G	0.006897	29	HDAC9	Genic	
93	rs3748816	1	2516606	C	−0.006724	61	MMEL1	Genic	
94	rs2219893	6	32877641	C	−0.006629	62	HLA-DOB	Intergenic	11329
95	rs138179	22	44523840	A	0.006614	41	ATXN10	Genic	
96	rs4468578	15	32192404	T	−0.006541	39	PGBD4	Intergenic	8621
97	rs17072702	4	183213759	G	0.006464	36	TENM3	Intergenic	268408
98	rs1035597	18	30741953	T	−0.006450	37	DTNA	Intergenic	17550
99	rs2346391	17	28343273	G	−0.006354	58	SPACA3	Genic	
100	rs2351643	8	63239550	A	−0.006308	44	NKAIN3	Intergenic	84636
101	rs693955	6	44299898	A	−0.006173	28	SLC29A1	Genic	
102	rs1464459	1	235911706	T	0.006142	45	RYR2	Genic	
103	rs4851610	2	102501084	G	0.006103	52	SLC9A4	Genic	
104	rs12639104	3	148900311	T	−0.006080	25	ZIC1	Intergenic	286282
105	rs11863993	16	5726576	G	−0.005952	48	ALG1	Intergenic	651692
106	rs4665072	2	160031937	T	−0.005922	50	BAZ2B	Genic	
107	rs1429248	2	155060456	T	−0.005912	25	GALNT13	Intergenic	45146
108	rs1133045	7	76762152	C	−0.005911	28	CCDC146	Genic	
109	rs673567	1	18150844	G	−0.005861	43	ACTL8	Intergenic	125242
110	rs1172990	9	92569628	A	−0.005845	36	SYK	Intergenic	76373
111	rs4652825	1	182059946	G	−0.005777	36	RGL1	Genic	
112	rs10009456	4	136314125	A	0.005765	53	PABPC4L	Intergenic	972500
113	rs1550305	3	117027353	T	0.005750	23	LSAMP	Genic	
114	rs6937046	6	165086231	C	0.005682	36	C6orf118	Intergenic	527304
115	rs7684552	4	16691100	T	0.005627	21	LDB2	Intergenic	181893
116	rs17761155	17	1380663	G	0.005579	18	PITPNA	Genic	
117	rs7205534	16	80759119	G	−0.005548	44	MPHOSPH6	Genic	
118	rs748832	3	16826206	G	−0.005533	37	PLCL2	Genic	
119	rs10237550	7	112845911	T	−0.005492	44	PPP1R3A	Intergenic	459102
120	rs3744647	17	8165001	T	−0.005424	48	ARHGEF15	Genic	

(continued)

#	RS	Chr	BP	Ref	Weight	Stab. (%)	GeneSymbol	Location	Dist. (bp)
121	rs13314993	3	32990473	G	0.005422	61	CCR4	Intergenic	19471
122	rs7937334	11	117696484	T	0.005386	32	CD3E	Intergenic	5016
123	rs7137478	12	83773349	T	−0.005359	36	SLC6A15	Intergenic	6192
124	rs753507	13	23329745	T	0.005314	31	MIPEP	Genic	
125	rs305522	3	11990313	C	0.005305	27	SYN2	Intergenic	30712
126	rs12361949	11	7152914	C	−0.005288	38	SYT9	Intergenic	77079
127	rs1945921	11	130467592	G	−0.005256	39	SNX19	Intergenic	176547
128	rs735890	12	1624685	G	0.005219	28	WNT5B	Genic	
129	rs6578296	11	2736546	G	−0.005209	20	KCNQ1	Genic	
130	rs4900384	14	97568704	G	0.005191	28	BCL11B	Intergenic	1141536
131	rs4369223	1	15400921	T	0.005184	38	TMEM51	Genic	
132	rs2837768	21	40951877	C	0.005149	33	DSCAM	Genic	
133	rs6890606	5	167830877	C	0.005101	37	WWC1	Intergenic	2276
134	rs10951781	7	44984788	T	−0.005097	29	MYO1G	Genic	
135	rs12685669	9	20112244	T	0.005088	26	MLLT3	Intergenic	224196
136	rs4663581	2	235992660	A	−0.005079	33	AGAP1	Intergenic	75409
137	rs17561086	11	105903230	T	−0.005077	26	GUCY1A2	Intergenic	160254
138	rs2658862	11	131076004	T	−0.005065	24	NTM	Genic	
139	rs9634310	12	45832324	T	0.005054	50	PCED1B	Genic	
140	rs6780338	3	71112098	G	−0.005048	19	FOXP1	Genic	
141	rs10967946	9	27451643	T	−0.005006	21	MOB3B	Genic	
142	rs355816	2	165417128	T	−0.004986	17	COBLL1	Intergenic	10351
143	rs12196758	6	90052018	G	−0.004933	20	GABRR2	Genic	
144	rs10854214	20	59137313	T	0.004924	36	CDH4	Intergenic	123651
145	rs1014486	3	161173806	G	−0.004914	43	IL12A	Intergenic	15731
146	rs11763166	7	131503450	C	0.004912	30	PLXNA4	Genic	
147	rs17685465	5	132767225	C	0.004912	29	FSTL4	Genic	
148	rs9384261	6	150593881	A	0.004882	15	PPP1R14C	Genic	
149	rs372741	18	75370277	G	−0.004860	40	NFATC1	Genic	
150	rs3810909	9	114971513	C	0.004833	20	FKBP15	Genic	
151	rs6679417	1	241147146	A	0.004826	13	CEP170	Intergenic	208902
152	rs690336	18	8844219	A	−0.004820	24	SOGA2	Intergenic	22380
153	rs1479552	3	2539452	C	0.004813	37	CNTN4	Genic	
154	rs2166488	2	157868082	T	−0.004698	21	GALNT5	Genic	
155	rs2611249	4	166825748	C	−0.004653	32	CPE	Intergenic	187535
156	rs218218	2	33181898	C	0.004639	24	LTBP1	Genic	
157	rs913908	13	29623876	A	−0.004617	26	KATNAL1	Intergenic	56800
158	rs9399642	6	148647729	G	0.004551	25	SASH1	Intergenic	58167
159	rs336506	2	105691151	C	0.004525	21	NCK2	Intergenic	146800
160	rs1864516	8	4352789	G	0.004520	27	CSMD1	Genic	

(continued)

#	RS	Chr	BP	Ref	Weight	Stab. (%)	GeneSymbol	Location	Dist. (bp)
161	rs347142	3	32452828	G	0.004519	21	CMTM7	Genic	
162	rs6845272	4	136759345	T	−0.004436	38	PABPC4L	Intergenic	1417720
163	rs7761698	6	149483938	G	0.004428	45	UST	Intergenic	46992
164	rs4450019	1	113065234	T	−0.004418	32	FAM19A3	Genic	
165	rs4858692	3	25050171	C	−0.004392	38	RARB	Intergenic	395055
166	rs2364482	12	6372392	G	−0.004390	16	LTBR	Intergenic	2027
167	rs494387	19	39256568	C	−0.004350	19	LSM14A	Intergenic	98819
168	rs6956744	7	82209312	A	0.004331	21	PCLO	Intergenic	16514
169	rs2901840	2	120844238	T	−0.004252	15	INHBB	Intergenic	20317
170	rs856135	1	157216129	G	0.004237	21	PYHIN1	Intergenic	2987
171	rs920572	3	144472022	A	0.004212	26	SLC9A9	Genic	
172	rs1553985	4	76773628	C	0.004111	33	CDKL2	Genic	
173	rs2837766	21	40950152	T	−0.004093	34	DSCAM	Genic	
174	rs4679208	3	127363812	C	0.004063	29	ALDH1L1	Genic	
175	rs9290242	3	165898590	A	−0.004053	20	SI	Intergenic	281253
176	rs8035542	15	91595305	T	−0.004039	21	RGMA	Intergenic	162139
177	rs1254930	14	61579400	C	−0.004032	38	SYT16	Genic	
178	rs9915813	17	45368114	A	−0.004006	16	DLX4	Intergenic	33717
179	rs2253612	2	201276678	C	0.003984	14	AOX1	Intergenic	33030
180	rs2253698	20	1493617	C	−0.003964	26	SIRPB1	Genic	
181	rs11221388	11	127980211	A	−0.003948	29	ETS1	Intergenic	31975
182	rs11110390	12	99399032	A	−0.003925	40	NR1H4	Genic	
183	rs9615482	22	46032213	C	−0.003924	22	TBC1D22A	Intergenic	84279
184	rs12509421	4	7980412	C	0.003907	25	AFAP1	Genic	
185	rs1581688	7	112510329	G	0.003903	17	GPR85	Genic	
186	rs11715416	3	194797310	G	0.003838	32	OPA1	Genic	
187	rs4890643	18	42167123	A	0.003806	29	RNF165	Genic	
188	rs11062040	12	1961518	A	0.003798	28	DCP1B	Genic	
189	rs1174746	7	53109485	A	0.003772	29	POM121L12	Intergenic	37735
190	rs705352	7	90536300	T	−0.003751	16	CDK14	Genic	
191	rs4912274	1	57903614	T	−0.003731	22	DAB1	Genic	
192	rs2290600	3	109586117	C	0.003689	43	MYH15	Genic	
193	rs7071424	10	131364237	C	−0.003684	16	MGMT	Genic	
194	rs1542287	16	8366510	C	−0.003671	34	METTL22	Intergenic	260513
195	rs7079743	10	115182472	T	0.003630	29	HABP2	Intergenic	120398
196	rs1540528	2	241767359	T	0.003617	17	PPP1R7	Genic	
197	rs9871790	3	39388528	T	0.003615	23	SLC25A38	Intergenic	11691
198	rs11646037	16	6176433	C	0.003608	19	RBFOX1	Genic	
199	rs4689915	4	4717231	C	0.003562	16	STX18	Intergenic	122638
200	rs10864210	1	214083474	G	0.003473	20	USH2A	Genic	

(continued)

#	RS	Chr	BP	Ref	Weight	Stab. (%)	GeneSymbol	Location	Dist. (bp)
201	rs10478	13	29675202	C	0.003471	19	KATNAL1	Genic	
202	rs2376997	20	29782860	A	−0.003448	20	BCL2L1	Intergenic	9177
203	rs4240702	9	136735680	T	−0.003422	33	COL5A1	Genic	
204	rs526282	12	248165	G	0.003396	24	SLC6A13	Intergenic	8685
205	rs2835930	21	38043371	A	0.003353	29	KCNJ6	Genic	
206	rs26435	5	165240985	A	0.003314	21	TENM2	Intergenic	1403435
207	rs1378942	15	72864420	G	0.003296	23	CSK	Genic	
208	rs3744700	17	4584759	T	−0.003241	23	CXCL16	Genic	
209	rs2882513	2	156334094	C	0.003216	24	NR4A2	Intergenic	556407
210	rs3117230	6	33183613	C	0.003146	93	HLA-DPB2	Intergenic	4660
211	rs4237270	9	70696279	C	−0.003144	17	PIP5K1B	Genic	
212	rs1565585	12	27037709	T	−0.003121	17	TM7SF3	Genic	
213	rs4233131	1	185960354	C	−0.003106	20	PLA2G4A	Intergenic	736090
214	rs4756856	11	16598294	G	0.003096	27	SOX6	Genic	
215	rs4937390	11	128285012	C	−0.003094	22	KCNJ5	Genic	
216	rs4238606	16	11435090	T	−0.003091	23	RMI2	Intergenic	82941
217	rs784678	9	108951910	C	0.003080	19	RAD23B	Intergenic	133821
218	rs7633774	3	23708196	A	0.003067	24	UBE2E2	Intergenic	101869
219	rs6664618	1	66487172	T	−0.003003	23	PDE4B	Genic	
220	rs8012823	14	72343297	A	−0.002981	11	DPF3	Genic	
221	rs4296166	14	32022118	A	−0.002937	24	AKAP6	Genic	
222	rs10120215	9	91490874	G	−0.002911	21	UNQ6494	Genic	
223	rs7863610	9	77416239	T	−0.002910	12	PCSK5	Intergenic	279678
224	rs1468791	2	102458453	A	0.002841	44	SLC9A4	Genic	
225	rs6054024	20	6177282	A	−0.002555	14	FERMT1	Intergenic	129080
226	rs2981479	3	126285921	C	0.002553	31	SLC12A8	Genic	
227	rs999915	1	85111666	A	−0.002398	18	LPAR3	Genic	
228	rs2835931	21	38043518	T	0.000359	5	KCNJ6	Genic	
