# Supplementary material for: Accurate and Robust Genomic Prediction of Celiac Disease Using Statistical Learning
Source: PLoS Genet. 2014 Feb 13;10(2):e1004137. doi: 10.1371/journal.pgen.1004137 (PMC3923679; doi:10.1371/journal.pgen.1004137)
Supplement: Table S2 — Summary of screening results at different prevalence levels for the combine dataset Finn+IT+NL dataset, using different cutoffs to declare the samples as disease cases (expressed as % of the population). The smaller the cutoff, the stricter the definition of a disease case. (PDF) [file pgen.1004137.s007.pdf]

Prevalence (%)	Threshold (% of population)	Sensitivity (%)	Specificity (%)	Correct positive diagnoses per 1000 positive diagnoses (1000×PPV)	Correct negative diagnoses per 1000 negative diagnoses (1000×NPV)	Incorrect diagnoses per 1000 correct disease diagnoses
1	1	9	100	193	991	4999
	2	16	99	161	992	6785
	3	23	99	149	992	6955
	5	30	97	99	993	10008
	10	49	93	67	995	14657
	15	62	89	54	996	17948
	20	74	85	45	997	21429
	30	85	75	33	998	30479
	40	92	65	25	999	39778
	50	95	54	20	999	50481
	60	96	43	16	999	61449
	70	97	33	14	999	72394
	90	99	11	11	999	94035
	95	100	5	10	1000	99350
3	1	8	100	426	973	1576
	2	15	99	374	974	1755
	3	21	99	342	976	2003
	5	30	97	256	978	2994
	10	46	93	173	983	4844
	15	60	89	148	987	5845
	20	72	85	127	990	6974
	30	85	75	96	994	9565
	40	92	65	74	996	12669
	50	95	54	59	997	15982
	60	97	43	50	998	19376
	70	98	33	42	998	22770
	90	99	11	33	998	29753
	95	100	5	31	997	31482
10	1	8	100	739	906	357
	2	15	99	689	912	454
	3	21	99	661	918	514
	5	29	97	554	925	808
	10	46	93	436	939	1298
	15	60	89	387	952	1586
	20	71	85	344	963	1909
	30	84	75	277	977	2618
	40	91	65	225	985	3452
	50	94	54	187	988	4351
	60	97	43	161	991	5233
	70	98	33	140	994	6141
	90	99	11	111	993	8030
	95	100	5	105	993	8496
20	1	8	100	860	812	163
	2	14	99	825	823	212
	3	20	99	808	833	238
	5	28	97	730	845	370
	10	45	93	628	872	592
	15	59	89	581	897	721
	20	71	85	538	920	859
	30	84	75	460	949	1176
	40	91	65	392	966	1552
	50	94	54	338	972	1959
	60	96	43	298	979	2355
	70	98	33	266	984	2763
	90	99	11	217	985	3604
	95	100	5	208	984	3813

Supplementary Table 2
