# Supplementary material for: Accurate and Robust Genomic Prediction of Celiac Disease Using Statistical Learning
Source: PLoS Genet. 2014 Feb 13;10(2):e1004137. doi: 10.1371/journal.pgen.1004137 (PMC3923679; doi:10.1371/journal.pgen.1004137)
Supplement: Text S1 — Supplementary Methods. (PDF) [file pgen.1004137.s008.pdf]

Accurate and Robust Genomic Prediction of Celiac Disease Using Statistical Learning

Supplementary Methods

Gad Abraham, Jason A. Tye-Din, Oneil G. Bhalala, Adam Kowalczyk, Justin Zobel, and Michael Inouye

November 7, 2013

1 The Genomic Risk Score

The model that produces the genomic risk score [1] is given in Supplementary Table 1*. Also provided is the intercept β_0. The intercept is not required if we are only interested in ranking patients according to their risk, and do not wish to compare the cutoffs with the results in the main manuscript. The final score for an individual is

$$\hat{y}_i = \beta_0 + \sum_{j=1}^{228} x_{ij}\beta_j, \qquad (1)$$

where x_{ij} is the genotype for the jth SNP in the ith sample.

The easiest way to produce a risk score for a dataset is using PLINK, as it will take care to use the correct minor allele. Assuming that the data are called `DATA` and are in BED/BIM/FAM format:

```
plink --noweb --score grs.txt --bfile DATA
```

which will produce a file named `DATA.profile`.

The profile file can be read into R, and the predictions written back to the file `profile.txt`:

```
d <- read.table("DATA.profile", header=TRUE)
intercept <- -0.757226
d$GRS <- d$SCORE * d$CNT + intercept
write.table(d[, c("FID", "IID", "GRS")],
   file="profile.txt", row.names=FALSE, col.names=FALSE)
```

Note that PLINK divides the profile score by the number of alleles, which we undo by multiplying by CNT.

*A PLINK-compatible text file `grs.txt` is available at `http://dx.doi.org/10.6084/m9.figshare.154193`.

2 Estimating Diagnostic Performance of HLA Typing

2.1 Population Estimates

We can estimate sensitivity (sens), specificity (spec), PPV, and NPV from the confusion matrix tabulating the true positives (TP), false positives (FP), true negatives (TN), and true positives (TP), as follows: Sens = TP / (TP + FN); Spec = TN / (TN + FP); PPV = TP / (TP + FP); NPV = TN / (TN + FN). For PPV and NPV, we use the observed prevalence in the data, hence PPV=precision here.

In the 1% of the population setting, assuming conservatively that 30% of the population are HLA positive, and that 99.6% of CD-positive individuals are positive for HLA ($= 0.996 \times 0.01 = 0.00996$ of the population are true positives), we derive the confusion matrix:

	HLA^+	HLA^-	
CD^+	TP = 0.00996	FN = 0.00004	True = 0.01
CD^-	FP = 0.29	TN = 0.7	False = 0.99
	Pos = 0.3	Neg = 0.7	Total = 1.00

From this matrix we estimate Sens = 0.996, Spec = 0.707, PPV = 0.033, and NPV = 1.

In the 10% prevalence setting, it has estimated that 73% of 1st-degree family members of an index case with celiac disease had HLA DQ2 [74], which we use as a proxy for overall HLA status due to the lack of individuals with DQ8 in that study and the fact that DQ2 carries most of the CD risk. Celiac disease was diagnosed in 11%, of which all had HLA DQ2. This leads to the confusion matrix:

	$DQ2^+$	$DQ2^-$	
CD^+	TP = 0.11	FN = 0.00	True = 0.11
CD^-	FP = 0.62	TN = 0.27	False = 0.89
	Pos = 0.73	Neg = 0.27	Total = 1.00

From this matrix we estimate Sens = 1, Spec = 0.3, PPV = 0.151, and NPV = 1.

2.2 HLA Imputation

HLA type information was not available for our data. To compare the estimates of PPV/NPV from the literature with our data, we used the R package HIBAG v1.2.0.1 [35] to impute the HLA type, based on SNPs in chr6. `HIBAG` uses ensemble classifiers to predict HLA haplotypes from genotypes, after having been trained on data with known haplotypes.

The output from `HIBAG` is in the form of two *DQA1* haplotypes and two *DQB2* haplotypes (alleles). Note that allele 1 and allele 2 are not phased, that is, allele 1 for *DQA1* is not necessarily on the same chromosome as allele 1 for *DQB1*. We then combined the alleles using the following rules to derive the imputed HLA-DQ2/DQ8/DQ2.5 heterodimers for each sample:

- DQ2.2: *DQA1*02:01 / DQB1*02:02*
- DQ8: *DQA1*03:01*, X / *DQB1*03:02*, Y
- DQ2.5 homozygous: *DQA1*05*, *05* / *DQB1*02*, 02
- DQ2.5 heterozygous: *DQA1*05*, X / *DQB1*02*, Y, where X and Y are any alleles (except for X=05 and Y=02 that would make the type DQ2.5 homozygous)

Note that for each rule, there may be several allele configurations that need to be tested due to missing phase information. For example, for DQ2.2 there are four configurations that could lead to the same observed heterodimer:

- *DQA1* allele 1 = 02:01, *DQB1* allele 1 = 02:02
- *DQA1* allele 2 = 02:01, *DQB1* allele 1 = 02:02
- *DQA1* allele 1 = 02:01, *DQB1* allele 2 = 02:02
- *DQA1* allele 2 = 02:01, *DQB1* allele 2 = 02:02

2.2.1 Presence/Absence of Imputed CD Risk Heterodimers

Current clinical practice in CD diagnosis is the use of DQ2.2 / DQ8 / DQ2.5 heterodimer status as a binary variable (presence/absence), for exclusion of CD in individuals with suspected CD. Applying the same logic as in Supplementary Section 2.1, we derived the confusion tables for the UK1 and UK2 datasets:

	UK1			UK2		
Pheno.	HLA+	HLA−		HLA+	HLA−	
CD+	TP = 0.3514	FN = 0.0023	True = 0.3536	TP = 0.2648	FN = 0.0077	True = 0.2725
CD-	FP = 0.3718	TN = 0.2745	False = 0.6464	FP = 0.4217	TN = 0.3058	False = 0.7275
	Pos = 0.7232	Neg = 0.2768	Total = 1	Pos = 0.6865	Neg = 0.3135	Total = 1

Assuming a prevalence of $K = 1\%$, we obtain PPV of 0.017 for both UK1 and UK2, and an NPV of 0.9998 and 0.9993 for UK1 and UK2, respectively. For prevalence of $K = 10\%$, PPV=16% and NPV=99% for both UK1 and UK2, largely in agreement with literature-based estimates (Supplementary Section 2.1)

3 Analysis of CD Predictive Performance

We compared the predictive performance of several methods based on SNPs, haplotypes, and combinations of SNPs and haplotypes (Supplementary Figure 2):

- SparSNP (ℓ_1-penalized SVM) on all autosomal SNPs (denoted *GRS All*).
- SparSNP on all MHC SNPs, defined as chr6 29.7Mb–33.3Mb (denoted *GRS MHC*).

- SparSNP on all non-MHC SNPs, defined as all autosomal SNPs outside the above MHC range (denoted *GRS non-MHC*).
- Unpenalized logistic regression on the heterodimer types inferred from the HLA haplotypes (using HIBAG), categorized into three groups: high risk (HLA-DQ2.5 homozygous or DQ2.2/DQ2.5), low risk (HLA-DQ2/DQ8 negative), and medium risk (all others) [21] (denoted *Romanos HLA*).
- The Romanos 3-level HLA approach together with a weighted risk score defined by 57 non-HLA Immunochip SNPs, with weights given in the original publication [21] (denoted *Romanos HLA+57 SNPs*). This was only available for the Immunochip dataset.
- Unpenalized logistic regression on a set of individual SNPs that tag known HLA haplotypes [36] (rs2395182, rs7775228, rs2187668, rs4639334, rs7454108, and rs4713586). For the UK2, Finn, IT, and NL datasets, 5 out of the 6 SNPs were present or had proxies with perfect LD ($r^2 = 1$). One remaining SNP, rs4639334, could not be tagged, but is a marker for HLA-DQ7, a low-risk haplotype for CD. For the UK1 dataset, three assayed SNPs were assayed (rs2395182, rs7775228, rs2187668) and were also in the UK2 dataset. This model was denoted *Monsuur HLA SNPs*.

For the comparison of the UK2→UK1 and UK2→Immunochip performance, we used the SNPs common to each pair of datasets, respectively, and excluded related individuals (see main Methods section). The SparSNP models were optimized using 10×10-fold validation on the UK2 dataset, and the best model was applied to the other datasets without any further tuning. For the Monsuur HLA SNPs and Romanos HLA methods, we used a similar approach were the model was trained using logistic regression on the UK2 dataset (we report cross-validation results), and externally validated on the other datasets. For the Romanos HLA + 57 SNPs, we used logistic regression in the Immunochip data, modeling both the HLA type and the risk score given by the non-HLA SNPs as in the original publication, within cross-validation.

4 Checking for Confounding by Population Stratification

We used `smartpca` from EIGENSOFT 4.2 [67] to estimate the top 10 principal components for the UK2 dataset. Prior to running PCA, we further reduced the QCd UK2 data to remove regions of known high LD or inversions [72]:

- Excluded regions: chr5: 44Mb–51.5Mb, chr6: 25Mb–33.5Mb, chr8: 8Mb–12Mb, chr11: 45Mb–57Mb.
- Thinned the SNPs by LD ($r^2 < 0.2$) using `plink --indep-pairwise 1500 150 0.2`.

In `smartpca`, we also used the option `nsnpldregress:5` to further account for LD. This left 98,983 autosomal SNPs from which the final PCs were determined.

First, we examined whether the top 10 PCs were themselves predictive of case/control status in cross-validation, using unpenalized logistic regression (`R` package `rms` [73]). The 10 PCs were essentially non-predictive of case/control status (average AUC=0.52 in 200 bootstrap replications).

Second, we trained L1-penalized models on the original UK2 dataset (after removing the outliers) together with the 10 PCs as covariables. The PCs were allowed to enter the model in the training phase, however, in testing the PCs were ignored, i.e., the final predictor is composed of SNPs only, so that any predictive information contained in the PCs will not contribute to the final estimates of AUC, and if the PCs are highly predictive (at the expense of the SNPs), this will manifest as low AUC, indicating strong confounding by population structure. On the other hand, if AUC remains high, this indicates that the SNPs successfully account for case/control status despite removing the population effects, and that population structure is not an important factor in the predictive power of this model. After cross-validation, we took the best SNP model, and externally validated it on the other datasets.

Supplementary Figure 5a shows the cross-validated AUC for the UK2 model accounting for the 10 PCs, showing essentially no difference in AUC between that model and the original model without the PCs. External validation is shown in Supplementary Figure 5b, demonstrating that the high predictive ability for the model accounting for the 10 PCs is conserved in the other datasets. Together, these results strongly indicate that population stratification does not play any substantial role in the high predictive ability of these models.

References

[72] J. Fellay, D. Ge, K. Shianna, S. Colombo, B. Ledergerber, E. Cirulli, et al. Common Genetic Variation and the Control of HIV-1 in Humans. *PLoS Genet.*, 5:e1000791, 2009.

[73] F. E. Harrell. *rms: Regression Modeling Strategies*, 2013. R package version 4.0-0.

[74] A. Rubio-Tapia, C. T. Van Dyke, B. D. Lahr, A. R. Zinsmeister, M. El-Youssef, S. B. Moore, M. Bowman, L. J. Burgart, L. J. Melton, and J. a. Murray. Predictors of family risk for celiac disease: a population-based study. *Clinical Gastroenterology and Hepatology*, 6(9):983–7, September 2008.
