# Supplementary material for: Correction: Accurate and Robust Genomic Prediction of Celiac Disease Using Statistical Learning
Source: PLoS Genet. 2014 Apr 14;10(4):e1004374. doi: 10.1371/journal.pgen.1004374 (PMC3986057; doi:10.1371/journal.pgen.1004374)
Supplement: File S2 — Republished corrected article. [file pgen.1004374.s002.pdf]

OPEN ACCESS Freely available online

PLOS GENETICS

Accurate and Robust Genomic Prediction of Celiac Disease Using Statistical Learning

Gad Abraham[1,2], Jason A. Tye-Din[3,4,5], Oneil G. Bhalala[1], Adam Kowalczyk[2], Justin Zobel[2], Michael Inouye[1]*

1 Medical Systems Biology, Department of Pathology and Department of Microbiology & Immunology, The University of Melbourne, Parkville, Victoria, Australia, **2** NICTA Victoria Research Lab, Department of Computing and Information Systems, The University of Melbourne, Parkville, Victoria, Australia, **3** The Walter and Eliza Hall Institute of Medical Research, Parkville, Victoria, Australia, **4** Department of Medical Biology, The University of Melbourne, Parkville, Victoria, Australia, **5** Department of Gastroenterology, The Royal Melbourne Hospital, Parkville, Victoria, Australia

Abstract

Practical application of genomic-based risk stratification to clinical diagnosis is appealing yet performance varies widely depending on the disease and genomic risk score (GRS) method. Celiac disease (CD), a common immune-mediated illness, is strongly genetically determined and requires specific HLA haplotypes. HLA testing can exclude diagnosis but has low specificity, providing little information suitable for clinical risk stratification. Using six European cohorts, we provide a proof-of-concept that statistical learning approaches which simultaneously model all SNPs can generate robust and highly accurate predictive models of CD based on genome-wide SNP profiles. The high predictive capacity replicated both in cross-validation within each cohort (AUC of 0.87–0.89) and in independent replication across cohorts (AUC of 0.86–0.9), despite differences in ethnicity. The models explained 30–35% of disease variance and up to ~43% of heritability. The GRS's utility was assessed in different clinically relevant settings. Comparable to HLA typing, the GRS can be used to identify individuals without CD with ≥99.6% negative predictive value however, unlike HLA typing, fine-scale stratification of individuals into categories of higher-risk for CD can identify those that would benefit from more invasive and costly definitive testing. The GRS is flexible and its performance can be adapted to the clinical situation by adjusting the threshold cut-off. Despite explaining a minority of disease heritability, our findings indicate a genomic risk score provides clinically relevant information to improve upon current diagnostic pathways for CD and support further studies evaluating the clinical utility of this approach in CD and other complex diseases.

Citation: Abraham G, Tye-Din JA, Bhalala OG, Kowalczyk A, Zobel J, et al. (2014) Accurate and Robust Genomic Prediction of Celiac Disease Using Statistical Learning. PLoS Genet 10(2): e1004137. doi:10.1371/journal.pgen.1004137

Editor: Joshua M. Akey, University of Washington, United States of America

Received August 1, 2013; **Accepted** December 8, 2013; **Published** February 13, 2014

Copyright: © 2014 Abraham et al. This is an open-access article distributed under the terms of the Creative Commons Attribution License, which permits unrestricted use, distribution, and reproduction in any medium, provided the original author and source are credited.

Funding: The celiac disease genotype data was generated under WTCCC award WT084743 and Coeliac UK funding. Part of this work utilized the computing resources of the Victorian Life Sciences Computation Initiative (project VR0126). This study makes use of data generated by the Wellcome Trust Case-Control Consortium. A full list of the investigators who contributed to the generation of the data is available from www.wtccc.org.uk. Funding for the project was provided by the Wellcome Trust under award 076113 and 085475. MI was supported by an NHMRC early career fellowship 637400. MI and GA were supported by University of Melbourne funding. JATD was supported by an NHMRC Postgraduate Medical Scholarship. This work was partially supported by the Australian Research Council, and by the NICTA Victorian Research Laboratory. NICTA is funded by the Australian Government as represented by the Department of Broadband, Communications, and the Digital Economy, and the Australian Research Council through the ICT Centre of Excellence program. This work was made possible through Victorian State Government Operational Infrastructure Support and Australian Government NHMRC IRIIS. The funders had no role in study design, data collection and analysis, decision to publish, or preparation of the manuscript.

Competing Interests: The authors have declared that no competing interests exist.

* E-mail: minouye@unimelb.edu.au

Introduction

Improving the diagnosis of celiac disease (CD), a common immune-mediated illness caused by dietary gluten, remains a clinical challenge [1,2]. Despite a prevalence of approximately 1% in most Western countries, lack of awareness and failure to implement appropriate serological, histological and genetic testing means that less than 30–40% of those affected by CD are diagnosed [1,3–5]. Undiagnosed CD is associated with reduced quality of life, substantial morbidity, and increased mortality, however, prompt diagnosis and treatment lowers the burden of disease and may reduce the rate of complications such as osteoporosis, autoimmune disease, and malignancy. Optimizing the diagnosis of CD is now recognized as an important goal for clinicians [6].

CD is characterized by a variable combination of gluten-dependent clinical manifestations, CD-specific antibodies and small bowel inflammation (villous atrophy) [7]. Traditional guidelines for the diagnosis of CD rely on demonstrating villous atrophy and improvement of symptoms, laboratory abnormalities, and/or small bowel inflammation upon exclusion of dietary gluten [8]. Current clinical practice is to screen for CD by detecting CD-specific serum antibodies and then confirm the diagnosis by undertaking small bowel biopsy to demonstrate typical villous atrophy. Serologic screening for CD with transglutaminase-IgA antibodies is reported to be highly sensitive and specific for CD (both >90%), imparting a high positive predictive value (PPV) of over 90% when assessing most populations [9,10], although the PPV can fall to 45–70% in community screening settings [11,12]. In practice, serological and histological assessments have technical limitations that generate both false negative and false positive diagnoses.

A key feature of CD is its strong dependence on the presence of susceptibility genes encoding for HLA DQ2.5, DQ8, and/or half the HLA DQ2.5 heterodimer (typically DQ2.2), seen in approximately 99.6% of all patients with CD [13]. These genes encode

Author Summary

Celiac disease (CD) is a common immune-mediated illness, affecting approximately 1% of the population in Western countries but the diagnostic process remains sub-optimal. The development of CD is strongly dependent on specific human leukocyte antigen (HLA) genes, and HLA testing to identify CD susceptibility is now commonly undertaken in clinical practice. The clinical utility of HLA typing is to exclude CD when the CD susceptibility HLA types are absent, but notably, most people who possess HLA types imparting susceptibility for CD never develop CD. Therefore, while genetic testing in CD can overcome several limitations of the current diagnostic tools, the utility of HLA typing to identify those individuals at increased-risk of CD is limited. Using large datasets assaying single nucleotide polymorphisms (SNPs), we have developed genomic risk scores (GRS) based on multiple SNPs that can more accurately predict CD risk across several populations in "real world" clinical settings. The GRS can generate predictions that optimize CD risk stratification and diagnosis, potentially reducing the number of unnecessary follow-up investigations. The medical and economic impact of improving CD diagnosis is likely to be significant, and our findings support further studies into the role of personalized GRS's for other strongly heritable human diseases.

immune-recognition molecules which facilitate CD4+ T cell recognition of specific gluten-derived peptides, a critical step in disease pathogenesis [14–18]. Recognizing the crucial role of these genes, the latest consensus diagnostic guidelines for CD recommend testing for these HLA heterodimers (HLA typing) as a first-line investigation for asymptomatic individuals identified at-risk of CD, such as 1st-degree relatives of an affected individual or those with suggestive symptoms [7]. However, a major flaw of HLA typing as a diagnostic tool is that a substantial proportion of the community, typically reported to be 30–40%, express HLA DQ2.5, DQ8, and/or DQ2.2, thus making the presence of these HLA types poorly predictive and of low specificity for CD [13]. Indeed, a recent Australian population study revealed that 56% of the community possessed at least one of these CD susceptibility haplotypes [5]. Thus, while HLA typing can exclude CD in the community with high confidence when the susceptibility haplotypes are absent, these haplotypes will be present in 30–56% of the population, the majority of whom would not have CD. Therefore, if assessed as a stand-alone test, HLA typing has exceptionally high sensitivity and negative predictive value (NPV) but very poor specificity and low positive predictive value (PPV) for CD. Since a positive result poorly predicts the presence of CD, HLA typing is not useful as a stand-alone diagnostic tool for CD. While the relative-risk for CD can be stratified based on the HLA subtype (CD risk DQ2.5>DQ8>DQ2.2) [19], these categories have low positive predictive value and do not provide clinically-informative attribution of CD risk [20]; HLA results are therefore interpreted as a binary outcome: CD susceptibility positive or negative. Despite these limitations, HLA typing is now widely utilized in clinical practice and typically determined using polymerase chain-reaction sequence specific oligonucleotide (PCR-SSO) hybridization, which is time and labor intensive, and costly (AU $120/sample, Medicare; in the USA cost varies but is typically US $150/sample or greater).

It is important to distinguish between three different approaches to analyzing the HLA region for association with CD. The first approach, currently in clinical practice, is HLA typing, as described above, where the HLA result is considered a binary variable and its utility is to exclude CD. A second approach, such as that taken by Romanos et al., utilizes the same HLA-DQ haplotypes, stratifies individuals into several nominal risk levels then fits a statistical model to empirically estimate the true risk in each group [21,22]. While HLA-DQ haplotypes may be inferred from typing several HLA SNPs, importantly the HLA SNPs are only used to assign the HLA type and the SNPs themselves are not directly modeled. The third approach, such as that used here, is based on direct concurrent modeling of many thousands of individual SNPs for association with CD in order to produce a more fine-grained predictive "genomic risk score" (GRS).

GRSs have been enabled by the advent of genome-wide association studies (GWAS), which perform unbiased testing of many thousands of SNPs for association with CD. Using GWAS, recent studies have identified multiple non-HLA SNP associations with CD [23,24]. GWAS are primarily concerned with the detection of variants associated with disease in order to gain insight into the disease etiology and genetic architecture. Due to the high number of significance tests, controlling for false positive associations is a major, valid concern. Therefore, SNP-based risk scores have tended to be constructed from the SNPs found to be significantly associated with the disease status [22,25]. However, due to the stringent multiple-testing corrections utilized in GWAS there may be other SNPs that fail to achieve genome-wide significance but may be predictive of disease status nonetheless and including them in the model could potentially result in higher predictive ability than achievable by models based solely on genome-wide significant SNPs. In contrast to the GWAS approach, the main overriding aim of a GRS from a clinical perspective is to achieve maximal predictive capacity, the inference of genetic architecture is secondary.

We have recently designed computational algorithms which efficiently fit L1-penalized multivariable classification models to genome-wide and whole-genome SNP data [26]. Such models were then shown to be preferable to several other methods such as the standard method of summing the per-SNP log odds (polygenic score) [27], mixed effects linear modeling [28,29], and unpenalized logistic regression, with both better precision for detecting causal SNPs in simulation and better case/control predictive power [30]. These advantages were consistent across several complex diseases, including two British studies of CD. However, the diagnostic implications of penalized models have not been previously examined nor has the robustness of such models in other populations or the advantage over HLA-typing approaches. In contrast to existing studies that examine a small number of genome-wide significant SNPs, we have shown that many more SNPs (potentially hundreds) are required to achieve optimal predictive ability for CD. Further, the standard GWAS approach of considering each SNP separately when estimating its effect size does not consider its correlation with other SNPs. We have shown that unpenalized predictive models based on these top SNPs suffer from lower predictive ability than L1-penalized models since the pre-screening introduces multiple highly correlated SNPs into the model, of which a substantial proportion may be redundant in terms of contribution to the predictive ability. Similar L1-penalized approaches have also recently been successfully applied to inflammatory bowel disease case/control Immunochip data, where models based on several hundred SNPs have led to high predictive ability [31].

Here, we provide a proof-of-concept that the GRS for CD, induced by L1-penalized support vector machine models, are able to achieve a predictive capacity and robustness that provides information not afforded by current diagnostic pathways utilizing

HLA typing alone. This GRS has the potential to provide greater clinical diagnostic utility by enabling each individual to be assigned a more informative risk score beyond the simple designation of "CD susceptible" or "CD non-susceptible", or "high risk" versus "low risk". To enable useful comparisons between diagnostic approaches, we model the GRS as a stand-alone test to "diagnose" CD, while at the same time acknowledging that real world clinical practice will need to draw upon clinical history, CD-specific serology and small bowel histology to confirm the diagnosis of CD. We assess the predictive power of the GRS both in cross-validation and in external validation, across six different European cohorts, showing that the models strongly replicate. We test our GRS on three other autoimmune diseases: type 1 diabetes, Crohn's disease, and rheumatoid arthritis, finding some predictive ability for T1D status but none for the others, thus largely supporting the specificity of the scores for CD. To overcome limitations of previous studies utilizing GWAS case/control studies, where ascertainment bias incurs substantially higher rates of false positive results, we undertake genomic prediction of CD in "real world" settings where the prevalence of CD is far lower and evaluate the performance of the GRS using PPV and NPV at several levels of CD prevalence. Unlike HLA typing, the GRS allows flexibility in determining who is considered at higher risk for CD by selecting a clinically determined user-specified threshold. We demonstrate how these scores can be practically applied at various prevalence levels to optimize sensitivity and precision. Finally, we show how the model can be calibrated to produce accurate predicted probabilities of disease.

Results

An overview of our analysis workflow is shown in Figure 1. We analyzed five CD datasets on the Illumina Infinium array platform: *UK1* and *UK2* (British descent), *Finn* (Finnish descent), *IT* (Italian descent), and *NL* (Dutch descent). We also utilized a dataset run on a fine-mapping array: *IMM* (Immunochip of British descent) (see Methods and Table 1). We have previously analyzed the AUC achievable in the UK1 and UK2 [30].

We trained L1-penalized support vector machines (SVM) [26] on the genotype data, including all post quality control (QC) autosomal SNPs unless otherwise indicated. These models are sparse models, (Methods) and varying the penalty induces models based on different number of SNPs with non-zero coefficients. We investigated the performance for various degrees of sparsity, and the models were fitted to all SNPs across the genome simultaneously. For each model's induced risk score, we estimated the area under the receiver operating characteristic curve (AUC) and the explained phenotypic variance [32], which depends on the assumed prevalence of disease.

Cross-Validation in Each Dataset

Within each dataset, we used 10×10 fold cross-validation to estimate the AUC and explained phenotypic variance on the liability scale. The explained phenotypic variance was derived from the AUC assuming a population prevalence of $K=1\%$. All cohorts showed high AUC in cross-validation (Figure 2a), with the Finnish and Italian cohorts having a maximum of 0.89, followed by the UK1 cohort (AUC = 0.88), and finally the UK2 and Dutch cohorts with a maximum AUC of 0.87. Both the UK1 and the Italian cohorts peaked at ~64 SNPs with non-zero weights, whereas the rest peaked at ~250 SNPs. Subsampling of the individuals in the UK2 dataset indicated diminishing returns with 80% of the sample size having the same AUC as 100% (Figure S1). Consistent with this, combining the UK1 and UK2 datasets did not increase AUC beyond UK2 alone (results not shown). It is also important to note that some of the control samples were population-based and were not explicitly screened for celiac disease, thus ~1% may be cryptic CD cases which potentially underestimates prediction performance in downstream analyses. These AUCs correspond to explained phenotypic variance of 30–35% (Figure 2b). Assuming a CD heritability of 80%, this translated to an explained genetic variance of 37–43%.

External Validation between Datasets

While cross-validation provides an estimate of the model's ability to generalize to unseen datasets, choosing the model with the highest AUC may lead to so-called "optimization bias" (also called "winner's curse") [33,34], potentially manifesting as lower performance in independent validation. Additionally, cross-validation cannot compensate for intra-dataset batch effects, as these would be present in both the training and testing folds, potentially artificially inflating the apparent predictive ability. To assess whether the models suffered from optimization bias and to control for the possibility of intra-dataset batch effects, we performed external validation. Based on the results of the cross-validation, we selected the best models trained on the UK2 dataset then, without any further tuning, tested them on the UK1, Finn, IT and NL datasets and computed the receiver-operating characteristic (ROC) curves (Figure 3a). Overall, the models trained on the UK2 cohort showed high reproducibility on the other cohorts, achieving AUC of 0.89–0.9 in the Finnish and UK1 datasets, indicating negligible optimization bias from the cross-validation procedure. We also examined the replication of different SNP sets (all autosomal, MHC, and non-MHC) trained on the UK2 dataset and tested on the others (Figure S2). The trends observed in cross-validation, namely similar performance for MHC and all autosomal SNPs and lower but still substantial performance of non-MHC SNPs, was observed in all external validation experiments.

Comparison of Genomic Risk Score with Methods Based on HLA Typing

Since HLA typing is commonly used for assessing CD risk status, we sought to compare the performance of an approach based on inferred HLA types with the GRS. We utilized the approach of Romanos et al. [21] on the Immunochip data, which relies on both HLA types and 57 non-HLA Immunochip SNPs (including one chrX SNP). Since directly measured HLA types were not available for our datasets, we imputed *HLA-DQA1* and *HLA-DQB1* haplotype alleles using HIBAG [35] and derived the presence of DQ2.2/DQ2.5-homozygous/DQ2.5-heterozygous/DQ8 heterodimer status. The coefficients for the HLA risk types in the HLA+57 SNP model were not available for the Romanos et al. method, thus we had to estimate these from our data. For application of the GRS method, we trained models on 18,309 autosomal SNPs from UK2 (the subset shared between the Illumina 670 and Immunochip) then externally validated these models on the Immunochip data. We trained three separate models: All autosomal SNPs, MHC SNPs, and autosomal non-MHC SNPs. As seen in Figure 3b, the GRS trained on either all SNPs or the MHC SNPs yielded higher AUC (0.87) than the Romanos HLA+57 SNPs (AUC = 0.85) or HLA type alone (AUC = 0.8). The predictive power of the GRS induced by SNPs outside the MHC was lower but still substantial at AUC = 0.72. We also performed similar analyses on the rest of the datasets (UK2 in cross-validation then externally validated on the rest), comparing the GRS with the HLA type and with analysis of HLA tag SNPs [36] commonly used to infer HLA types since the 57

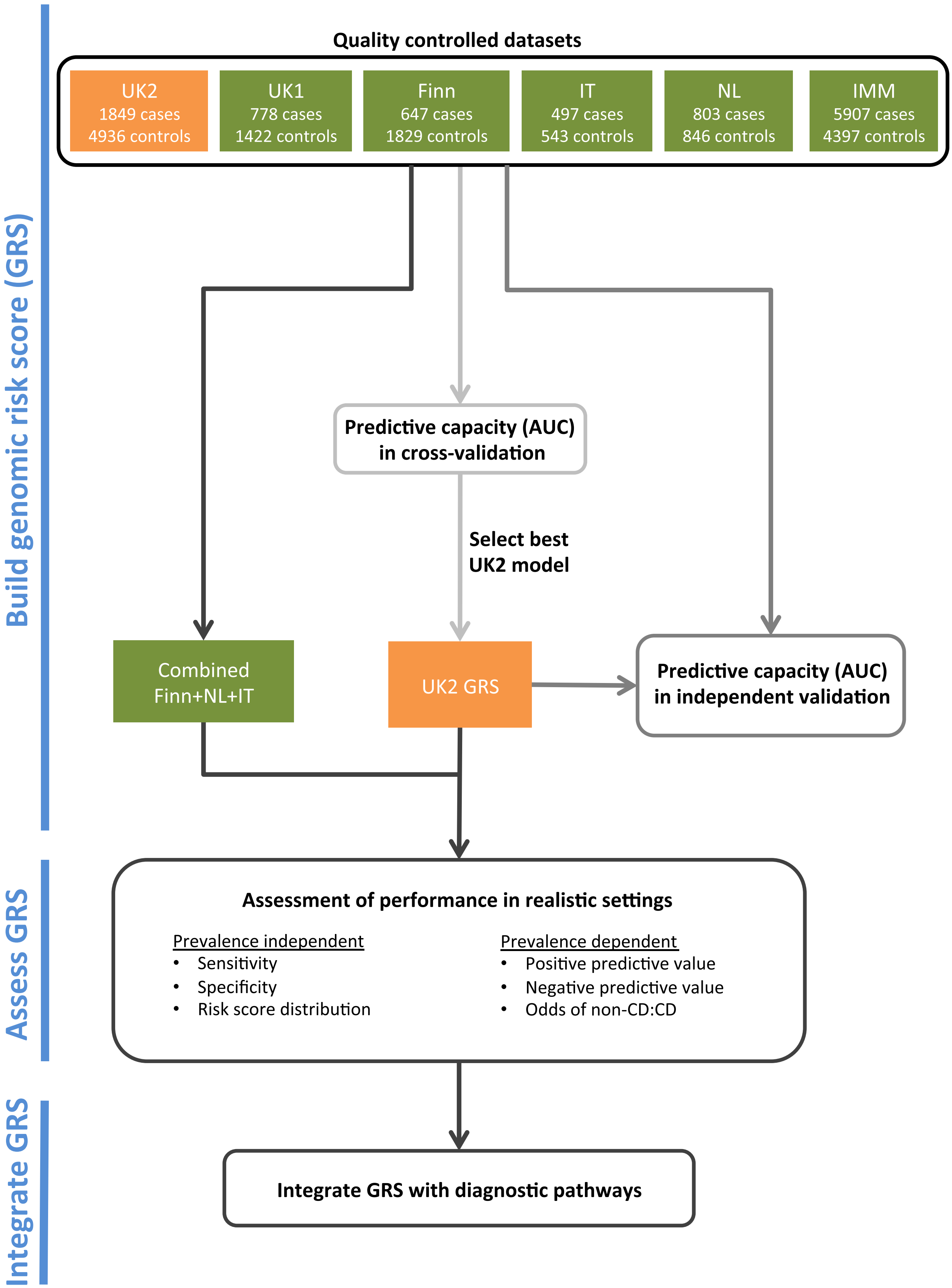

Figure 1. The analysis workflow.
doi:10.1371/journal.pgen.1004137.g001

Table 1. List of celiac disease datasets used in this study.

Name	Ethnicity	Platform	Autosomal SNPs post-QC	Male	Female	Cases	Controls	Total samples post-QC
**Finn**	Finnish	Illumina	513,952	1206	1270	647	1829	2476
**IT**	Italian	Illumina	515,641	332	708	497	543	1040
**NL**	Dutch	Illumina	515,169	752	897	803	846	1649
**UK1**	British	Illumina	301,659	938	1262	778	1422	2200
**UK2**	British	Illumina	515,444	2954	3831	1849	4936	6785
**IMM**	British	Immunochip	18,252*	3927	6377	5907	4397	10,304

*only SNPs in common with the post-QC UK2 dataset were analysed and are thus shown here.
doi:10.1371/journal.pgen.1004137.t001

non-HLA SNPs used by Romanos were not available on these platforms. As shown in Figure S2, the HLA type approach had consistently lower AUC (0.795–0.86) than analysis of the individual HLA tag-SNPs of Monsuur (AUC of 0.85–0.876, directly modeled using logistic regression on the SNPs in the UK2 then tested on the other datasets) and substantially lower than the GRS (AUC of 0.86–0.894).

Overall, our results showed that the L1-penalized SVM approach which modeled the SNPs directly was able to extract more information from the HLA region than the coarse-grained HLA haplotype model, either with or without the addition of the 57 non-HLA SNPs. This resulted in a gain in explained phenotypic variance of 3.5% over the best Romanos et al model in the Immunochip data.

Specificity of the Genomic Risk Score

We investigated whether the models of CD were predictive of case/control status in other immune-mediated diseases, specifically type 1 diabetes (T1D), rheumatoid arthritis (RA), and Crohn's Disease/Inflammatory Bowel Disease (Crohn's) from the WTCCC [37]. We utilized the 76,847 post-QC SNPs that appeared on both the UK2 Illumina and WTCCC Affymetrix 500K arrays. Despite the substantial reduction in the number of SNPs from the original data, we observed only small reductions in AUC in the restricted UK2 dataset in cross-validation, indicating that most of the predictive information was retained in the reduced SNP set (AUC = 0.85 at ~200 SNPs). The models trained on the UK2 were subsequently tested on the T1D, RA and Crohn's datasets. We also used the Finnish CD dataset as external validation to ensure that the high predictive performance observed in cross-validation on UK2 was replicated on other CD datasets and not degraded by using fewer SNPs. Overall, the models showed some predictive ability of T1D (AUC = 0.69), consistent with previous findings showing shared genetics between T1D and CD [38,39] (see Figure S3 for results for the MHC and non-MHC SNPs in T1D) but displayed very low performance (AUC 0.51–0.54) on the RA and Crohn's datasets. In contrast, performance on the Finnish CD cohort was only slightly lower (AUC = 0.85)

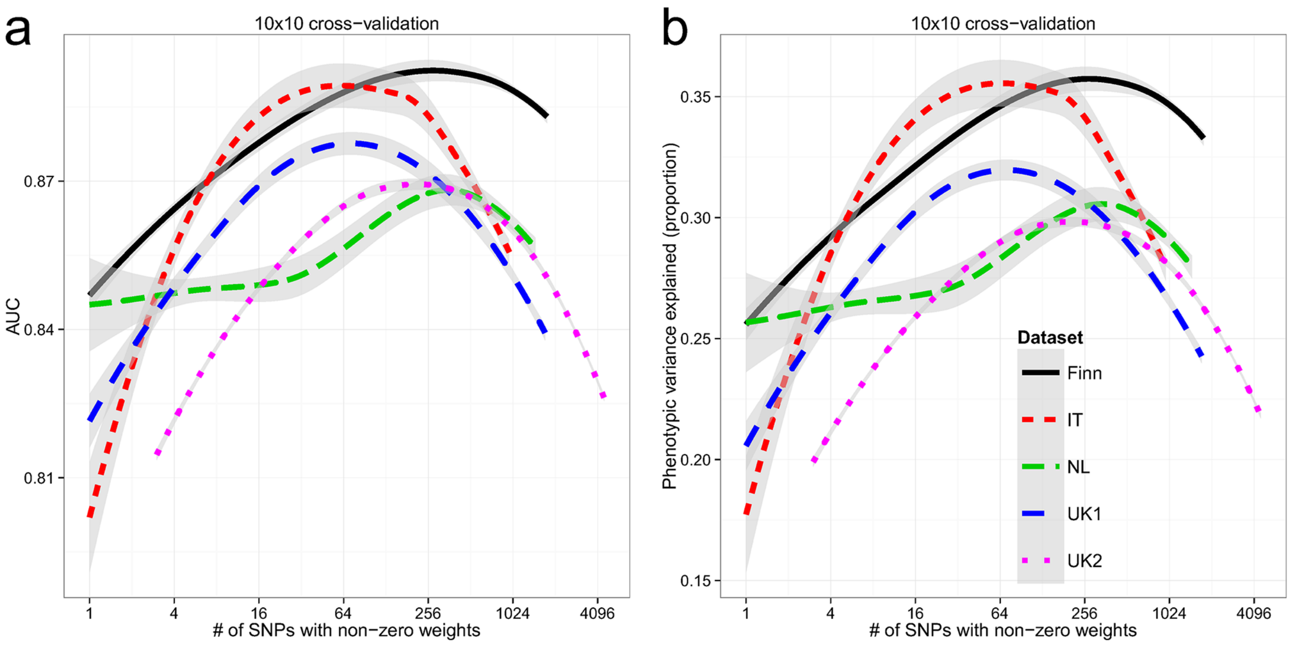

Figure 2. Building genomic models predictive of celiac disease. LOESS-smoothed (a) AUC and (b) phenotypic variance explained, from 10×10 cross-validation, with differing model sizes, within each celiac dataset. The grey bands represent 95% confidence intervals about the mean LOESS smooth.
doi:10.1371/journal.pgen.1004137.g002

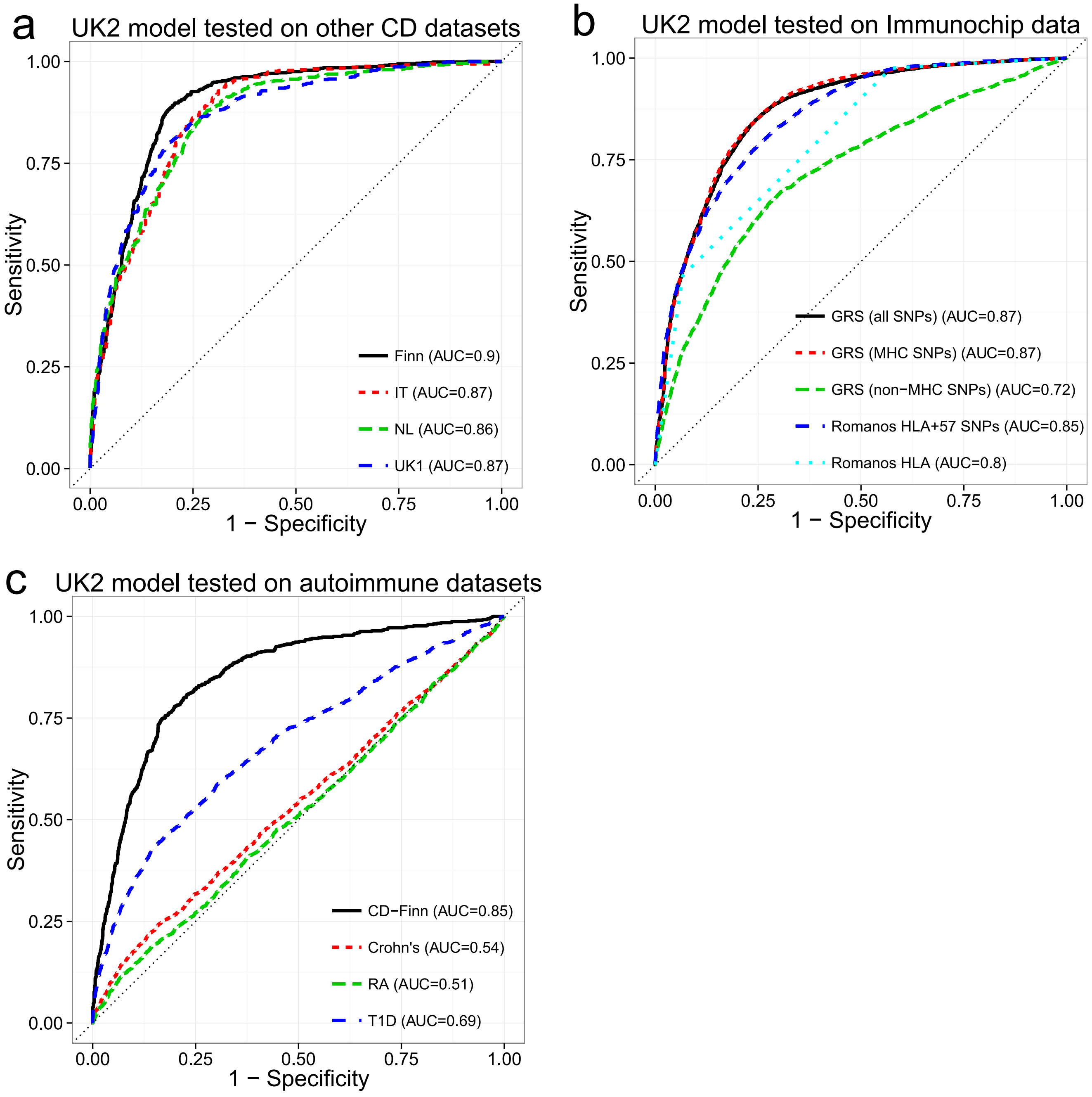

Figure 3. Performance of the genomic risk score in external validation, when compared to other approaches, and on other related diseases. ROC curves for models trained in the UK2 dataset and tested on (a) four other CD datasets, (b) the Immunochip CD dataset, comparing the GRS approach with that of Romanos et al. [21], and (c) three other autoimmune diseases (Crohn's disease, Rheumatoid Arthritis, and Type 1 Diabetes). We did not re-tune the models on the test data. For (b) and (c), we used a reduced set of SNPs for training, from the intersection of the UK2 SNPs with the Immunochip or WTCCC SNPs (18,252 SNPs and 76,847 SNPs, respectively). In (c), the same reduced set of SNPs was used for the CD-Finn dataset, in order to maintain the same SNPs across all target datasets.
doi:10.1371/journal.pgen.1004137.g003

compared with the full SNP set, again confirming that the CD models replicated across ethnic cohorts despite using a reduced set of SNPs (Figure 3c).

Analysis of a Combined Dataset

All CD datasets showed consistently high AUC both in cross-validation and in external validation, indicating that the risk of substantial confounding of the case/control status by ethnic cohort (via population stratification or strong intra-cohort batch effects) was low. Therefore, in order to increase statistical power in comparing the performance of the models, we created a combined dataset consisting of the Finnish, Dutch, and Italian cohorts, totaling 5158 samples (1943 cases and 3215 controls, 512,634 SNPs). This combined dataset is likely more representative of a real screening scenario where individuals of different ethnicities are being screened for CD. Figure 4a shows kernel density

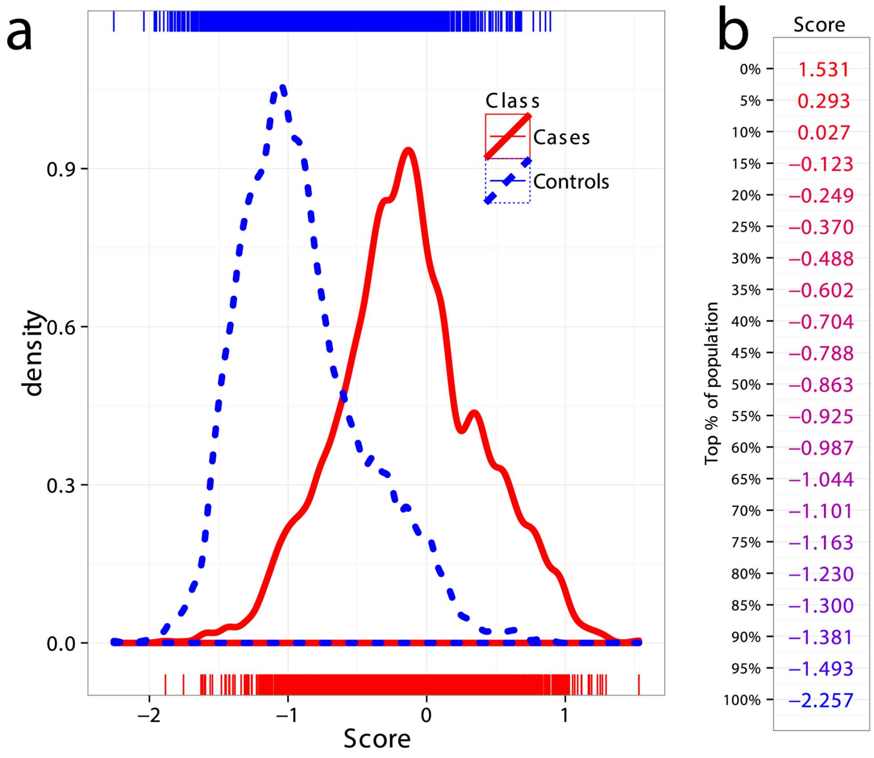

Figure 4. Distribution of genomic risk scores in cases and controls. (a) Kernel density estimates of the risk scores predicted using models on UK2 and tested in the combined dataset Finn+NL+IT, for cases and controls. (b) Thresholds for risk scores in terms of population percent, with the top more likely to be a CD and the bottom more likely to be non-CD.
doi:10.1371/journal.pgen.1004137.g004

estimates of the predicted risk scores for cases and controls in the combined dataset, where the scores are based on models trained on the UK2 dataset as previously described. As expected from the high AUC, there is substantial separation between the score distributions for the two classes. Also shown is the percentage of the combined population corresponding to a range of GRS thresholds (Figure 4b).

Positive and Negative Predictive Values under Different Prevalence Settings

The prevalence of CD in the general population, here taken to be 1%, is much lower than the prevalence in the case/control datasets where the cases are substantially over-represented owing to the study design. Considering the prevalence as the prior probability of a person having the disease (without knowing their genetic profile), then unless the likelihood of disease given the genotype is high as well, the posterior probability of disease will remain low. To quantify the predictive performance of our models while accounting for the prevalence, we estimated the precision of our models trained on UK2 on the Finn+NL+IT combined dataset. We down-sampled the cases in the combined dataset to simulate settings with different CD prevalence levels (1%, 3%, 10%, and 20%) and estimated precision and sensitivity in the test data, repeated in 50 independent simulations for each prevalence level (Figure 5a). The precision here is equivalent to the PPV [40] as the precision is estimated in data with the same prevalence as assumed by the PPV. The PPV is the posterior probability of having the disease given a positive diagnosis, and the NPV is the posterior probability of not having the disease given a negative diagnosis (a perfect model offers PPV = NPV = 1). Note that the lowest NPV achievable is 1−prevalence which translates to seemingly high NPV values in the low-prevalence setting, rendering NPV less useful for assessing classifiers in such settings as even a weak classifier can achieve apparently high NPV.

Population screening for CD is not currently accepted practice. Most evidence supports an active case-finding strategy where patients with risk-factors for CD, and therefore higher pre-test probability of CD than the population-wide average, are identified by their primary practitioner and screened. For example, the prevalence of CD in patients with a first-degree relative with CD is 10% or higher [41,42], and the prevalence of CD in patients with T1D ranges from 3–16% [43]. The increased CD prevalence in these groups of patients improves the apparent 'diagnostic' performance of the GRS. To examine the effect of prevalence on PPV, we first employed the GRS in a population-based setting (prevalence of 1%) which resulted in a PPV of ~18% at a threshold that identified 20% of the CD cases, but this dropped to ~3% at a threshold identifying 85% of the CD cases. In contrast, performance in more clinically relevant settings with higher CD prevalence was substantially better. For instance, the PPV increased from ~18% at 1% prevalence to ~40% at 3% prevalence, and to ~70% at 10% prevalence, with the sensitivity setting at 20% (Figure 5a). At 10% prevalence, increasing the GRS sensitivity to 60% resulted in a PPV of 40%, and at a sensitivity of 80% the PPV was ~30%. There were small differences in the AUC between prevalence levels, on the order of 1–3%, however all settings maintained high AUC at ≥0.86 (Figure 5b). Since sensitivity and specificity are independent of prevalence, these differences are likely due to the small number of cases in the low-prevalence settings and stochastic variations in the data caused by randomly sampling cases from different ethnicities, as each ethnicity showed slightly different predictability of CD in external validation,

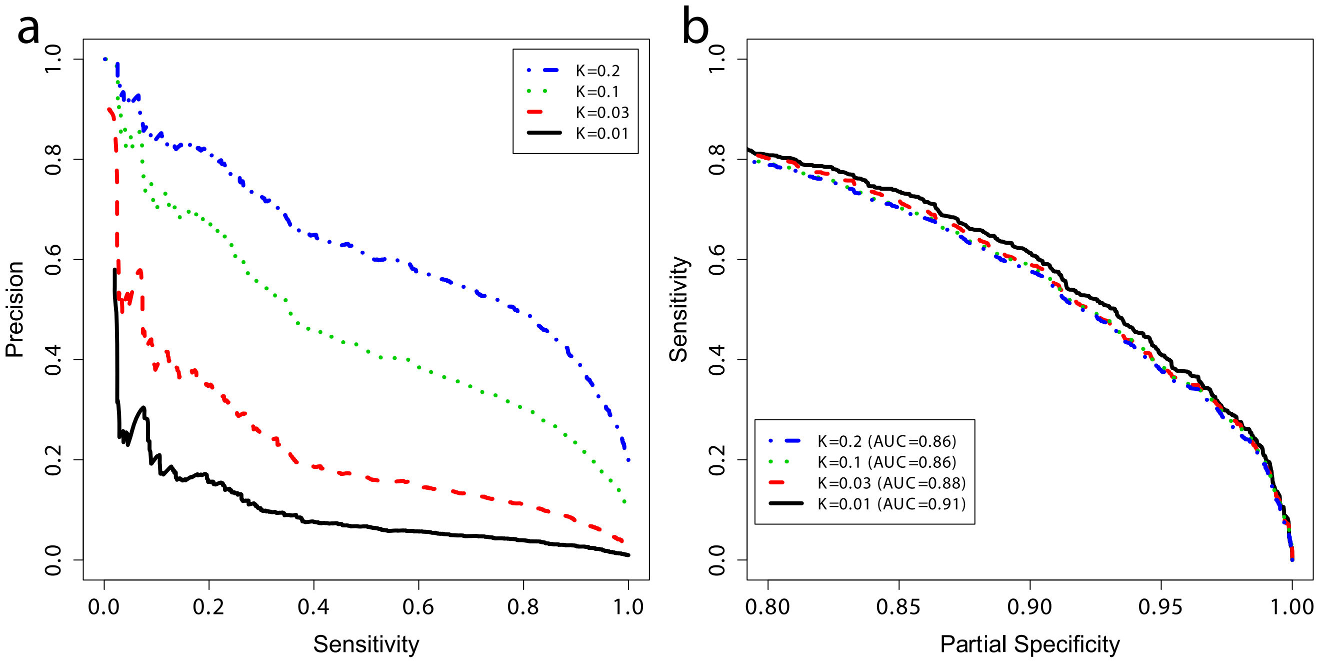

Figure 5. Performance at different prevalences and partial ROC curves. (a) Positive and negative predictive values and (b) partial ROC curves for models trained on UK2 using 228 SNPs in the model, and tested on the combined Finn+NL+IT dataset. *K* represents the prevalence of disease in the dataset and the curves are threshold-averaged over 50 replications. Note that precision is not a monotonic function of the risk score. Precision is equivalent to PPV here. A prevalence of ~10% corresponds to prevalence in first-degree relatives of probands with CD [42].
doi:10.1371/journal.pgen.1004137.g005

together with clinical heterogeneity resulting from different numbers of cryptic cases in the controls of each cohort.

Non-Disease Cases Implicated per True Disease Case

Another way to quantify the usefulness of predictive models as diagnostic tools is to evaluate the number of subjects without CD that are incorrectly identified as potential CD cases per each true CD diagnosis, and to do so at different levels of clinical risk (prevalence). This measure is equivalent to the posterior odds of not having CD given the genotypes $(1-\mathrm{PPV})/\mathrm{PPV}$, where a lower number is better (fewer incorrect cases implicated per true CD case). Figure 6 shows that at a sensitivity threshold to detect 20% of CD cases, the odds of incorrectly implicating CD were ~7:1 at prevalence of 1%, but this decreased to ~1:2 and ~1:5 at a prevalence of 10% and 20%, respectively. Further, at 10% CD prevalence the odds of incorrectly implicating CD were less than 1:1 while maintaining sensitivity of more than 30%, and for 20% CD prevalence up to 80% of true CD cases could be detected with such odds.

Application of the Genomic Risk Score

The application of the GRS is straightforward: once the SNPs in the model have been genotyped for a given patient, the GRS can be easily computed as the sum of the SNPs weights times the allele dosages plus an intercept term (Methods and Table S1). Our models consist of ~200 SNPs, hence the score can be easily computed in a spreadsheet or with PLINK. Whereas the models are fixed in the training phase, the interpretation of the scores depends on the screening setting in which they are used since selection of different risk thresholds leads to different false positive and false negative rates. In other words, the same numerical risk score may be interpreted differently in each setting, depending on the performance criteria required by the clinician, such as a minimum level of sensitivity or a maximum number of non-CD implicated per true CD implicated.

Figure 7 illustrates how the GRS could be applied in two commonly encountered but different clinical settings to (i) exclude individuals at average (background) risk of CD with high confidence, or to (ii) stratify individuals at higher risk of CD for further confirmatory testing. In the first setting, in order to optimize the NPV, a suitably low GRS threshold is selected, leading to a relatively large proportion of the population being considered as potentially at-risk of CD. An NPV of 99.6% (comparable to HLA testing) can be achieved at the population-wide 1% prevalence by setting a threshold corresponding to designating 15% of the population as CD cases (PPV of 5%). In the second setting, we modeled a scenario where the risk of CD is increased (for instance in patients with suggestive symptoms or clinical conditions) and risk stratification is sought to identify the patients most likely to benefit from further definitive investigation for CD. The prevalence of CD in those with higher-risk symptoms is approximately 3% [3,44] and in first-degree relatives of CD patients it is 10% [41,42]. In this second setting, we highlight two extreme choices of threshold as an example of what is achievable using the GRS at each prevalence level. The first threshold is stringent, predicting only a small number of high-confidence individuals as likely to have CD and subsequently leading to low sensitivity but higher PPV. The second threshold is low, implicating a larger number of individuals as likely to have CD and leading to higher sensitivity at the expense of reduced PPV.

More detailed results for a range of prevalence levels are shown in Table S2. These consider different cutoffs of the risk score expressed as a proportion of the population implicated for CD. We used the proportion of the population rather than proportion of the cases (sensitivity) to select risk thresholds

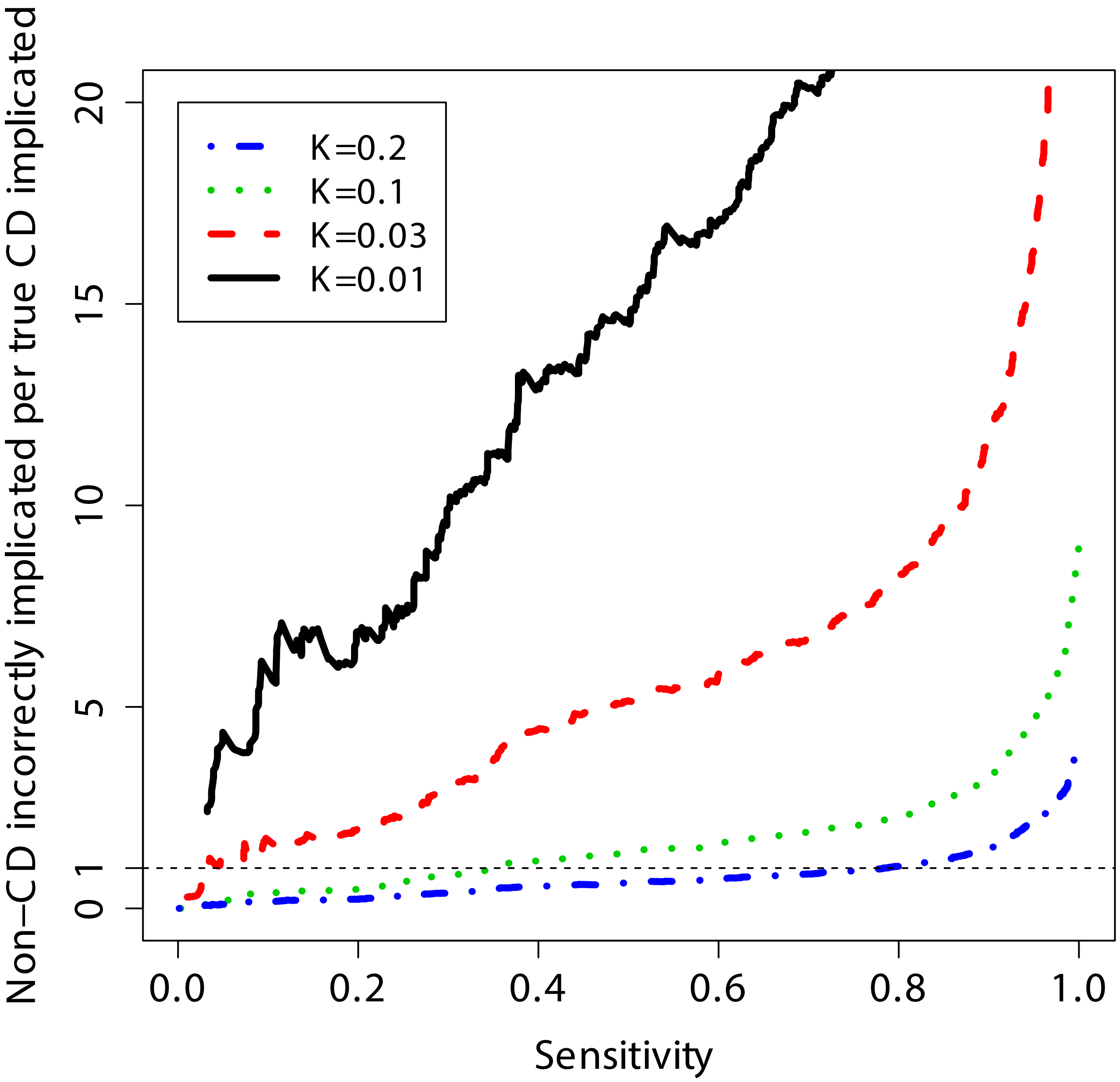

Figure 6. Clinical interpretation as a function of threshold and prevalence. The number of non-CD cases "misdiagnosed" (wrongly implicated by GRS) per true CD cases "diagnosed" (correctly implicated by GRS), for different levels of sensitivity. The risk score is based on a model trained on the UK2 dataset, and tested on the combined Finn+NL+IT dataset. The results were threshold-averaged over 50 independent replications. Note that the curve for $K=1\%$ does not span the entire range due to averaging over a small number of cases in that dataset.
doi:10.1371/journal.pgen.1004137.g006

since the true number of cases is unknown and we must select how to classify a given individual based only on their score relative to the population scores estimated in our data (Figure 4). As expected, sensitivity and specificity remain unchanged between the prevalence levels using the same risk score cutoff, however PPV, NPV and consequently the number of people incorrectly implicated as CD for each true CD case, depend strongly both on the prevalence and on the cutoff. Therefore, at a given prevalence level a suitable risk score cutoff can be selected in order to balance the two competing requirements of increasing the number of people correctly identified as having CD per true cases (PPV) while maintaining an acceptable level of sensitivity (coverage of the cases). A major benefit of the GRS is its flexibility in adapting to the appropriate clinical scenario and needs of the clinician. The PPV of the GRS can be adjusted up or down by varying the GRS cutoff and considering the acceptable level of sensitivity to detect CD. In practice, the most clinically appropriate cut-off thresholds would ideally be determined in local populations by undertaking prospective validation studies utilizing the GRS (See Discussion).

Risk Score Calibration

While the raw GRS cannot strictly be interpreted as the probability of disease given the genotypes, since it hasn't been normalized to be between 0 and 1, the score can be transformed

Excluding Celiac Disease
Aim: *optimizing NPV.*
We select GRS thresholds leading to NPV≥99.6%.

Clinical Setting	Prev (%)	Non-CD implicated per CD implicated	Sensitivity (% cases captured)	PPV (%)
**Population-wide**	**1**	**18:1**	**62**	**5**
Symptomatic	3	13:1	92	7
High-risk	10	6:1 +	98	14

Patient Stratification
Aim: *optimizing PPV.*
We show two GRS thresholds for each clinical setting.

Clinical Setting	Prev (%)	Non-CD implicated per CD implicated	Sensitivity (% cases captured)	PPV (%)
Population-wide	1	5:1	9	19
		50:1	95	2
Symptomatic	3	2:1	8	43
		16:1	95	6
**High-risk**	**10**	**1:4**	**8**	**74**
		**4:1**	**94**	**19**

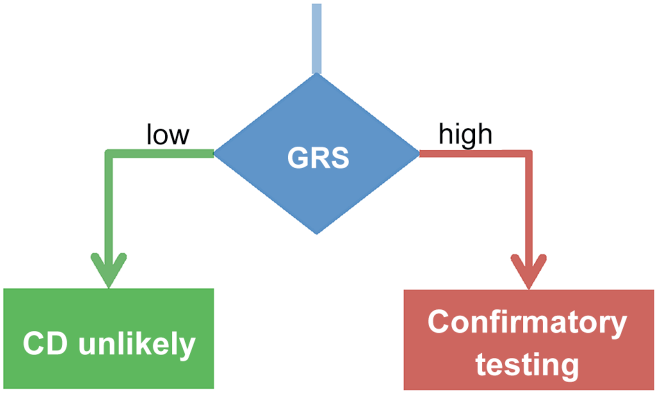

Figure 7. Example clinical scenarios. The GRS can be employed in different clinical scenarios and tuned to optimize outcomes. The GRS can be employed in a comparable manner to HLA testing (left table) to confidently exclude CD. In this scenario, we selected a GRS threshold based on NPV = 99.6% however a range of thresholds can be selected to achieve a high NPV (see note below). The GRS can also stratify CD risk (right table). Confirmatory testing (such as small bowel biopsy) would be reserved for those at high-risk. In this example, we present two scenarios: optimization of PPV or of sensitivity. In comparison to the GRS, all HLA-susceptible patients will need to undergo further confirmatory testing for CD. For more information on GRS performance across a range of thresholds, see Table S2. Prospective validation of the GRS in local populations would enable the most appropriate settings for NPV, PPV and sensitivity to be identified which provide the optimal diagnostic outcomes. + The highest achievable NPV at 10% prevalence was 99.4%.
doi:10.1371/journal.pgen.1004137.g007

into a probability using the empirical distribution of scores in the data (Figure 4). To assess the agreement between the predicted probability of disease and the observed probability of disease, we used calibration plots [45] that compared the predicted 5% quantiles of the risk scores, derived from models trained on the UK2 dataset and externally applied to the other datasets, with the observed probability of cases in each bin. For a well-calibrated GRS, the proportion of cases to samples in each bin should be approximately equal to the predicted risk. To correct for potential lack of calibration, we fitted a LOESS smooth to the calibration curve, which was then used to adjust the raw predictions into calibrated predictions. To avoid biasing the calibration step and to assess how well it performed in independent data, we randomly split each external dataset (Finn, IT, NL, and UK1) into two halves of approximately equal size. We assessed calibration in the first half of each dataset and fitted a LOESS smooth to the calibration curve (Figures S4a and S4c). We then used the LOESS smooth to calibrate the predictions for the other half of each dataset and assessed the calibration there (Figures S4b and S4d). Since the calibration is affected by prevalence, we assessed this procedure both in the observed data (prevalence of ~40%) and in a subsampled version with prevalence of ~10%. Overall, our calibration procedure was able to correct for a substantial amount of mis-calibration in the raw scores, even in the more challenging case of 10% prevalence.

Discussion

In this study, we have sought to exploit the strong genetic basis for CD and leverage comprehensive genome-wide SNP profiles using statistical learning to improve risk stratification and the diagnosis of CD. Our models showed excellent performance in cross-validation and were highly replicable in external validation across datasets of different ethnicities, suggesting that the genetic component is shared between these European ethnicities and that our models were able to capture a substantial proportion of it.

Importantly, even without explaining a majority of CD heritability, the models were robust and accurate, showing that it is not necessary to explain most of the heritability in order to produce a useful model.

The most frequently employed tools to diagnose CD are serology and small bowel histology, but both have limitations. Differences in the sensitivity of antibody recognition of commercially employed CD-specific antigens such as tissue transglutaminase, deamidated gliadin peptides, and endomysial antigen, as well as the human operator performing the assay can all influence findings and affect reproducibility of serological testing [9,46–49]. Serologic testing in children is reported to be less reliable before the age of 4 and up to 50% of children normalize elevated antibodies over time [50,51]. While small bowel histology remains the 'gold standard' confirmatory test, it is dependent upon patients willing and available to undergo endoscopy, adequate sampling by the gastroenterologist, and appropriate pathological processing and interpretation [52–54]. The frequencies of false positives and false negatives in CD serology assays vary widely and also partly depend upon what degree of histologic inflammation is considered compatible with CD [52,54–58]. Notably, the accuracy of both serologic and histologic testing for CD is dependent on the ongoing consumption of gluten. It is clear that clinically significant variability exists in serologic and histologic work-up for CD and new tools to improve the accuracy of CD diagnosis would be of benefit to clinicians. Given the strong genetic basis for CD, genomic tools are logical and appealing because they are relatively robust and less subject to the kind of variability seen with serologic and histologic assessment, are independent of age, and do not rely on dietary intake of gluten.

A major shortcoming of clinical HLA typing for risk prediction of CD is its poor specificity. HLA testing would result in virtually all CD cases detected but at the cost of approximately 30–56 people incorrectly implicated for each true case of CD. A significant advantage of the GRS approach is that it can be adapted to the clinical scenario in order to maximize PPV and diagnostic accuracy. By promoting accurate clinical stratification, the GRS could reserve invasive and more expensive confirmatory testing for those who would most likely benefit from further investigation to secure a diagnosis, and it would avoid unnecessary procedures in those who are HLA susceptible but unlikely to have CD. This provides both clinical and economic benefits. HLA typing does not provide the flexibility afforded by the GRS and cannot be effectively employed to identify those who would benefit from endoscopy. For instance, if HLA typing were used as a guide for further investigations, at 10% CD prevalence it would generate over five unnecessary endoscopies per correct endoscopy and at 1% CD prevalence it would generate 30–56 unnecessary endoscopies. Small bowel endoscopy is not a trivial undertaking – the procedure is costly (approximately AUD $750–$1000 for the procedure and associated pathology), has potential complications, necessitates a full day off work, and many patients are reluctant to undergo it.

The GRS can be used to exclude patients unlikely to have CD with a performance comparable to HLA typing. Testing with these parameters may be useful in the clinical scenario of assessing individuals at average risk of CD. A common example would be when a person has commenced a gluten-free diet prior to assessment for CD by serology or small bowel examination and are unwilling or unable to resume oral gluten intake in order to make testing reliable. This is an increasingly common clinical dilemma as the number of people following a gluten-free diet without adequate initial testing for CD continues to rise. In the United States approximately 30% of the adult population are interested in cutting back or avoiding dietary gluten [59].

The GRS can also be used to stratify the risk for CD in patients who present with suggestive clinical features. These risk factors include having a first-degree relative with CD or problems such as recurrent abdominal pain, bloating, diarrhea or constipation, fatigue, weight loss, unexplained anemia, autoimmune disease (including thyroid disease, T1D, autoimmune hepatitis, rheumatoid arthritis, and Sjogren's syndrome), infertility or early-onset osteoporosis [3,60]. Supporting the recently revised diagnostic guidelines for CD, which promote HLA testing as the 1st line investigation for higher-risk cases, genetic testing of CD is likely to be more informative in these sub-populations exhibiting higher-than-normal prevalence. While clinical guidelines recommend screening for CD in these high-risk populations [61], testing often poses a diagnostic dilemma as serologic assessment alone cannot confidently exclude a diagnosis, especially given the higher pre-test probability. HLA typing is not particularly informative as the CD HLA susceptibility haplotypes HLA-DQ2.5 and DQ8 are commonly present (manifesting in over 90% of patients with T1D and in 65% in first-degree relatives of individuals with CD) [62,63]. Stratifying these higher-risk patients based on a GRS will allow improved identification of those where small bowel biopsy is likely to be informative. Thus, a GRS should reduce the number of unnecessary small bowel biopsies in first-degree relatives who carry HLA susceptibility for CD but do not have it. We have found that our CD models had only moderate predictive ability for T1D, which is consistent with previous findings showing some shared genetics between T1D and CD [38]. Despite the substantial overlap of genetic factors for autoimmune disease, the CD models had negligible predictive ability for Crohn's disease and rheumatoid arthritis. These results indicate that our GRS is specific to CD and less likely to incorrectly identify patients with other autoimmune diseases as having CD, but further work is required to determine whether CD can be as confidently predicted in individuals with T1D as it is in non-T1D populations.

Another major clinical challenge that may benefit from genomic risk prediction is determining the natural history of potential CD (formerly termed 'latent CD') when there is serologic but not histologic evidence of CD, and identifying which patients are more likely to develop overt CD with small bowel inflammation [64]. Current practice is to follow-up all patients with immunologic evidence of gluten intolerance in order to capture those who will eventually develop overt disease. An analogous clinical scenario is that of children with positive CD serology, of whom 50% will fail to develop small bowel changes consistent with CD during follow-up [50,51]. In both clinical situations, it is reasonable to expect that a GRS can improve risk stratification of such patients for developing overt CD. Of course, environmental factors are important in the development of CD and the exact extent to which environmental versus genetic factors contribute to the development of overt CD remains unknown. Long-term follow-up studies of patients with potential CD will be necessary to establish the role of genomic risk prediction in this important subgroup.

Future work will look at optimizing our GRS as a tool to predict CD risk. Validation of our model in real-life practice will be important to confirm the clinical benefit of the GRS in conjunction with serology and/or over HLA typing alone, as well as to what extent other clinical predictors such as sex, age, and family history can contribute to clinically relevant risk prediction. Future prospective studies will enable direct optimization of

clinical utility (accuracy, practicality, throughput and cost) afforded by the GRS, for example in conjunction with CD serology. These studies will also provide a rigorous evidence base for suggested clinical guidelines of GRS usage. Importantly, appropriate GRS cut-off levels to maximize diagnostic accuracy (optimal PPV and NPV for each given clinical scenario and CD prevalence) could be obtained by local prospective validation. Such studies can identify the ultimate clinical role for the GRS: whether it can effectively replace HLA typing and also whether it is a stand-alone test or one to accompany CD serology. Hadithi et al showed that in patients at high-risk of CD the addition of HLA typing to CD serology had the same performance as either testing strategy alone [65], but the greater precision of the GRS over HLA typing may better complement CD serology. Understanding where the GRS fits in the diagnostic algorithm to optimize precision and cost-effectiveness will be essential, as is the role it might play in the diagnostic work-up of CD in populations with lower levels of clinical risk. Health economic modeling will address the cost-benefits of using the GRS in the diagnosis of CD, taking into account the cheaper cost of GRS over HLA typing, and include the downstream benefits of potentially reducing endoscopies (substantial cost savings and value to patients from reduced discomfort) as well as potential improvements in quality of life from the detection of CD.

Further, it may be that other statistical modeling approaches yield improvements in predictive power, for example non-additive models that consider epistatic interactions between SNPs. Another avenue for improvement is considering each CD subtype separately, recognizing potentially different genetic bases for these conditions. Based on our results, we do not expect substantial improvements from increasing sample size alone, however this will be important for adequately powered studies of lower frequency genetic variants of assumedly greater effect size.

In summary, this study demonstrates that simultaneous modeling of all SNPs using statistical learning was able to generate genomic risk scores that accurately predict CD to a clinically relevant degree. This was despite the models explaining only a minority of disease heritability. The GRS better enables clinicians to stratify patients according to their risk of CD compared to HLA typing alone and, we predict, more accurately determines those suitable for confirmatory testing in the form of small bowel biopsy. Reserving this invasive, time consuming and costly procedure for higher-risk cases is likely to improve the accuracy, cost and public acceptance of testing for CD, and by extension, benefit the overall diagnosis of CD in the community. By better prioritizing higher-risk patients for confirmatory testing, genomic risk prediction carries promise as a clinically useful tool to add to the clinician's diagnostic armamentarium. Ultimately, we envisage a clinical scoring algorithm based on the combination of clinical features, serologic, and genetic information that will accurately predict people with biopsy-confirmed CD and perhaps ultimately overcome the reliance on small bowel histology altogether. Further, the costs of genotyping a select number of marker SNPs with a low-plex, high throughput technology are already far lower than the costs of full HLA typing, resulting in a test that is cheaper, more flexible and more precise than HLA typing. More generally, this study demonstrates that statistical learning approaches utilizing SNPs can already produce useful predictive models of a complex human disease using existing genotyping platforms assaying common SNPs and suggests that similar approaches may yield comparable results in other complex human diseases with strong genetic components.

Methods

Ethics Statement

All participants gave informed consent and the study protocols were approved by the relevant institutional or national ethics committees. Details given in references van Heel et al [23] and DuBois et al [24]. All data was analysed anonymously.

Data

We analyzed six CD datasets: *UK1* [23], *UK2*, *IT*, *NL*, and *Finn* [24], and *IMM* [66]. The main characteristics of the datasets are listed in Table 1. In addition we used three WTCCC datasets (T1D, Crohn's, and RA) that have been described elsewhere [30,37]. UK1 used the Illumina Hap330v1-1 array for cases and Hap550-2v3 for controls, UK2 used the Illumina 670-QuadCustom-v1 for cases and 1.2M-DuoCustom-v1 for controls, the NL and IT datasets used the Illumina 670-QuadCustom-v1 in both cases and controls, and the Finn dataset used the Illumina 670-QuadCustom-v1 for cases and Illumina 610-Quad for controls. The WTCCC data (T1D, Crohn's, and RA) used the Affymetrix 500K array. In all of our models, we used autosomal SNPs only, and did not include the gender as a covariable, as models built separately on the two genders using the same sample size and case:control balance showed very similar performance in cross-validation on the UK2 dataset (results not shown). For analyses of the MHC region, we defined the MHC as all SNPs on chr6 in the range 29.7 Mb–33.3 Mb.

Quality Control

For each of the UK1, UK2, IT, NL, and Finn datasets, we removed non-autosomal SNPs, SNPs with MAF$<$1%, with missingness $>$1%, and those with deviations from Hardy-Weinberg Equilibrium in controls $P<5\times10^{-6}$. We also removed samples with missingness $>$1%. We tested identity-by-descent between samples in UK1 and UK2 and removed one of a pair of samples with pi-hat $\geq$0.05 (either between the datasets or within the datasets). The QC for the IMM Immunochip data has been previously described [66]; we estimated 5763 Immunochip samples to have pi-hat $\geq$0.125 (PLINK IBS) with any UK2 sample, and those were removed, leaving 10,304 Immunochip samples in total, with 18,252 SNPs shared with the UK2 dataset (post-QC). The QC for the WTCCC data (T1D, Crohn's, and RA) has been previously described [30,37].

Assessment of Population Structure Effects

To assess the impact of potential cryptic population structure, we estimated the top 10 principal components (PCs) for the UK2 with EIGENSOFT 4.2 [67], after removal of regions with high LD (see Text S1 for details). The principal components themselves showed almost no predictive ability (AUC = 0.52), and models trained on all SNPs accounting for these PCs showed indistinguishable performance from the non-adjusted model, both in cross-validation on the UK2 dataset and in external validation on the Finn, NL, and IT datasets (Figure S5), demonstrating that confounding of our UK2 models by population structure was negligible and was not a contributing factor to the high predictive ability.

Statistical Analysis

We used L1-penalized support vector machines (SVM) implemented in the tool SparSNP [26] (https://github.com/gabraham/SparSNP) as the classifiers. The L1-penalized SVM is a sparse linear model, that is, many or most of the SNPs will receive zero weight in the model, as determined by the L1

penalty. The use of a sparse model fits with our prior expectation that in autoimmune disease most SNPs will not be associated with disease status. The inherent sparsity of the model obviates the need for subsequent filtering of SNPs by weight, in order to decide which ones show strong evidence of association and which are spurious, as would be required in a non-sparse (L2-penalized) model. In addition, in extensive simulation and in analysis of real genotype data, including the two celiac disease datasets UK1 and UK2, we have previously shown the advantage of L1-penalized SVMs over commonly used approaches such as polygenic scores (sum of the log odds), linear mixed models (GCTA), and unpenalized logistic regression [30]. The advantage of sparse models over standard linear mixed models in predicting autoimmune disease has been recently confirmed in type-1 diabetes as well [68]. We have also shown that our L1-penalized SVMs achieved essentially identical performance to L1-penalized logistic regression (glmnet) in cross-validation over the Finnish subset of the celiac disease dataset, while being substantially faster [26]. Unlike single marker approaches that estimate the effect size of each SNP separately, the L1-penalized SVM is a multivariable model, where the estimated effect of each SNP is conditional on all other SNPs, thereby implicitly accounting for the linkage disequilibrium (LD) between SNPs. Besides imposing sparsity, the L1 penalty tends to produce models where one representative SNP is selected out of a group of highly correlated SNPs, while the rest remain with a zero weight, in contrast with L2-penalized or unpenalized models where many or all of these SNPs may receive a non-zero weight. For an in-depth discussion of these issues and the effects of varying LD levels on the performance of multivariable models, see [30].

The L1-penalized SVM model is induced by minimizing the L1-penalized squared-hinge loss over N samples and p SNPs,

$$L(\beta_0,\beta)=\frac{1}{2N}\sum_{i=1}^{N}\max\{0,1-y_i(x^T{}_i\beta+\beta_0)\}^2+\lambda\sum_{j=1}^{p}|\beta_j|$$

where x_i is the p-vector of genotypes for the ith sample in allele-dosage coding $\{0, 1, 2\}$, y are the binary phenotypes $\{-1, +1\}$, β is the p-vector of weights, β_0 is the intercept (also called the bias, which is not penalized), and λ is the L1 penalty. We also investigated adding an L2 penalty to the model (elastic-net), however, based on initial cross-validation experiments, we found no advantage in the L2 penalty and subsequently did not use it. All of our models were additive in the allele dosage $\{0, 1, 2\}$.

The genomic risk score $\hat{y}_i$ for a new sample x_i consisting of p genotypes is then

$$\hat{y}_i=\beta_0+\sum_{j=1}^{p}x_{ij}\beta_j$$

where the continuous value $\hat{y}_i$ is later thresholded at different values to produce a binary predicted class. The model was evaluated over a grid of penalties, in 10-fold cross-validation, repeated 10 times. The optimal number of SNPs in the model was decided based on the model with the highest average AUC across the replications. The final model was a consensus model, averaged over all $10\times10=100$ models, and containing approximately the number of SNPs determined earlier. Post processing and plotting of the results was performed in R [69], together with the package ggplot2 [70].

Measures of Predictive Performance

To quantify the predictive performance of the models in cross-validation and external validation, we employed receiver operating characteristic (ROC) curves (sensitivity versus 1 minus specificity), the area under the ROC curve (AUC) [71], and the proportion of phenotypic variance explained [32].

To quantify predictive performance in different population settings, we used the positive and negative predictive values, which can be estimated as

$$PPV=\frac{sens\times prev}{sens\times prev+(1-spec)\times(1-prev)}$$

and

$$NPV=\frac{spec\times(1-prev)}{spec\times(1-prev)+(1-sens)\times prev}$$

where "sens" is the sensitivity = TP/(TP+FN), "spec" is the specificity = TN/(FP+TN), and "prev" is the population prevalence. The PPV/NPV are equivalent to the posterior probability of a person having/not having the disease given a positive/negative diagnosis, respectively. When the PPV and precision are estimated in data with identical prevalence (that is, the observed prevalence in the data is identical to the prevalence in the population for which we wish to estimate PPV), they are equivalent. Precision is defined as TP/(TP+FP).

Supporting Information

Figure S1 LOESS-smoothed AUC in 10×10-fold cross-validation for the random subsamples of the UK2 dataset, in increasing sample size proportions of the original data (n = 6785).
(EPS)

Figure S2 Results of externally validating the predictive models, trained on UK2 in cross-validation, and tested on the other CD datasets. Legend: *Romanos HLA*: 3-levels of risk (low, medium, high) [21] based on imputed HLA type (HIBAG); *Romanos HLA+57 SNPs* (Immunochip only): 3-level HLA risk plus 57 Immunochip non-HLA SNPs [21]; *Monsuur HLA SNPs*: logistic regression on individual HLA SNPs [36] (5/6 SNPs or proxies thereof were found in the UK2/Finn/NL/IT datasets, 3/6 were found in the subset of UK1 shared with UK2); *GRS MHC SNPs*: SparSNP run on individual SNPs on chr6 within 29.7 Mb–33.3 Mb; *GRS non-MHC SNPs*: SparSNP run on individual autosomal SNPs outside MHC; *GRS all SNPs*: SparSNP run on all autosomal SNPs.
(EPS)

Figure S3 ROC curves for CD model trained on SNP subsets of the UK2 dataset that were assayed for the WTCCC-T1D dataset: *All SNPs* (76,847 SNPS), *MHC SNPs* (186 SNPs in the MHC region of chr6, 29.7–33.3 Mb), and *Non-MHC SNPs* (76,661 SNPs outside the MHC).
(EPS)

Figure S4 Calibration plots, comparing predicted score in 5% quantiles against observed proportions of cases falling within the bin. The score comes from models trained on the UK2 dataset, and tested on the rest of the datasets. The bars show 95% confidence intervals using the Agresti-Coull method for proportions. We randomly split the test datasets into two halves. In the first half, we plotted the original quantiles of the scores and fitted a LOESS smooth to them. We did this for the original case/control data (prevalence of 40%), shown in (a), and for a

subsampled version of the data with prevalence of 10% (c). We then used the LOESS smooth to correct the original quantiles, forming a calibrated score, one for each dataset (Finn, IT, NL, UK1), which was then applied to the second half of the data, shown in (b) and (d) for prevalence of 40% and 10% respectively. The second half of the data was not used in the calibration step.
(EPS)

Figure S5 (a) LOESS-smoothed AUC from 10-fold cross-validation for the UK2 model (all autosomal SNPs), accounting for the top 10 PCs (included in training but not in testing). (b) External validation of the best UK2 model that accounted for the PCs (PCs excluded from testing).
(EPS)

Table S1 The predictive model. The SNPs are sorted in decreasing order of the absolute value of their model weight averaged over the 10×10 cross-validation folds. Stability is the percentage of times a SNP was selected to have non-zero weight over the 10×10-cross-validation folds. Intercept: −0.757226. To annotate the SNPs we used Bioconductor 2.12 together with the packages VariantAnnotation 1.6.5 and TxDb.Hsapiens.UCSC.hg18.knownGene 2.9.0. We considered a SNP to be genic if it was annotated to fall inside one of the regions {spliceSite, intron, fiveUTR, threeUTR, coding, promoter} and intergenic otherwise. For intergenic SNPs, we also annotate the nearest gene and the distance to it. All positions are in hg18 coordinates.
(PDF)

Table S2 Summary of screening results at different prevalence levels for the combine dataset Finn+IT+NL dataset, using different cutoffs to declare the samples as disease cases (expressed as % of the population). The smaller the cutoff, the stricter the definition of a disease case.
(PDF)

Text S1 Supplementary Methods.
(PDF)

Acknowledgments

We thank the investigators of the van Heel et al., 2007, Dubois et al., 2010 and Trynka et al., 2011 papers (David van Heel, Cisca Wijmenga, and Lude Franke) for providing the celiac disease data.

Author Contributions

Conceived and designed the experiments: MI GA. Performed the experiments: GA. Analyzed the data: GA MI OGB JZ AK. Contributed reagents/materials/analysis tools: GA JZ AK JATD. Wrote the paper: GA MI JATD.

References

1. Anderson RP (2011) Coeliac disease is on the rise. Med J Aust 194: 278–279.
2. Green PH, Cellier C (2007) Celiac disease. N Engl J Med 357: 1731–1743.
3. Catassi C, Kryszak D, Louis-Jacques O, Duerksen DR, Hill I, et al. (2007) Detection of Celiac disease in primary care: a multicenter case-finding study in North America. Am J Gastroenterol 102: 1454–1460.
4. Dube C, Rostom A, Sy R, Cranney A, Saloojee N, et al. (2005) The prevalence of celiac disease in average-risk and at-risk Western European populations: a systematic review. Gastroenterology 128: S57–67.
5. Anderson RP, Henry MJ, Taylor R, Duncan EL, Danoy P, et al. (2013) A novel serogenetic approach determines the community prevalence of celiac disease and informs improved diagnostic pathways. BMC Med 11: 188.
6. Thompson T (2005) National Institutes of Health consensus statement on celiac disease. J Am Diet Assoc 105: 194–195.
7. Husby S, Koletzko S, Korponay-Szabo IR, Mearin ML, Phillips A, et al. (2012) European Society for Pediatric Gastroenterology, Hepatology, and Nutrition guidelines for the diagnosis of coeliac disease. J Pediatr Gastroenterol Nutr 54: 136–160.
8. Walker-Smith J, Guandalini S, Schmitz J, Shmerling D, Visakorpi J (1990) Revised criteria for diagnosis of coeliac disease. Report of Working Group of European Society of Paediatric Gastroenterology and Nutrition. Archives of Disease in Childhood 65: 909–911.
9. Hill ID (2005) What are the sensitivity and specificity of serologic tests for celiac disease? Do sensitivity and specificity vary in different populations? Gastroenterology 128: S25–32.
10. Hill PG, Forsyth JM, Semeraro D, Holmes GK (2004) IgA antibodies to human tissue transglutaminase: audit of routine practice confirms high diagnostic accuracy. Scand J Gastroenterol 39: 1078–1082.
11. Chin MW, Mallon DF, Cullen DJ, Olynyk JK, Mollison LC, et al. (2009) Screening for coeliac disease using anti-tissue transglutaminase antibody assays, and prevalence of the disease in an Australian community. Med J Aust 190: 429–432.
12. Leffler DA, Schuppan D (2010) Update on serologic testing in celiac disease. Am J Gastroenterol 105: 2520–2524.
13. Karell K, Louka AS, Moodie SJ, Ascher H, Clot F, et al. (2003) HLA types in celiac disease patients not carrying the DQA1*05-DQB1*02 (DQ2) heterodimer: results from the european genetics cluster on celiac disease. Human Immunology 64: 469–477.
14. Henderson KN, Tye-Din JA, Reid HH, Chen Z, Borg NA, et al. (2007) A structural and immunological basis for the role of human leukocyte antigen DQ8 in celiac disease. Immunity 27: 23–34.
15. Karell K, Louka AS, Moodie SJ, Ascher H, Clot F, et al. (2003) HLA types in celiac disease patients not carrying the DQA1*05-DQB1*02 (DQ2) heterodimer: results from the European Genetics Cluster on Celiac Disease. Human Immunology 64: 469–477.
16. Lundin KE, Scott H, Fausa O, Thorsby E, Sollid LM (1994) T cells from the small intestinal mucosa of a DR4, DQ7/DR4, DQ8 celiac disease patient preferentially recognize gliadin when presented by DQ8. Human Immunology 41: 285–291.
17. Lundin KE, Scott H, Hansen T, Paulsen G, Halstensen TS, et al. (1993) Gliadin-specific, HLA-DQ(alpha 1*0501,beta 1*0201) restricted T cells isolated from the small intestinal mucosa of celiac disease patients. Journal of Experimental Medicine 178: 187–196.
18. Tye-Din JA, Stewart JA, Dromey JA, Beissbarth T, van Heel DA, et al. (2010) Comprehensive, quantitative mapping of T cell epitopes in gluten in celiac disease. Sci Transl Med 2: 41ra51.
19. Megiorni F, Mora B, Bonamico M, Barbato M, Nenna R, et al. (2009) HLA-DQ and risk gradient for celiac disease. Human immunology 70: 55–59.
20. Sollid LM, Lie BA (2005) Celiac disease genetics: current concepts and practical applications. Clin Gastroenterol Hepatol 3: 843–851.
21. Romanos J, Rosen A, Kumar V, Trynka G, Franke L, et al. (2013) Improving coeliac disease risk prediction by testing non-HLA variants additional to HLA variants. Gut [Epub ahead of print].
22. Romanos J, van Diemen CC, Nolte IM, Trynka G, Zhernakova A, et al. (2009) Analysis of HLA and non-HLA alleles can identify individuals at high risk for celiac disease. Gastroenterology 137: 834–840, 840.e831–833.
23. van Heel DA, Franke L, Hunt KA, Gwilliam R, Zhernakova A, et al. (2007) A genome-wide association study for celiac disease identifies risk variants in the region harboring IL2 and IL21. Nature Genetics 39: 827–829.
24. Dubois PCA, Trynka G, Franke L, Hunt Ka, Romanos J, et al. (2010) Multiple common variants for celiac disease influencing immune gene expression. Nature Genetics 42: 295–302.
25. Izzo V, Pinelli M, Tinto N, Esposito MV, Cola A, et al. (2011) Improving the estimation of celiac disease sibling risk by non-HLA genes. PloS ONE 6: e26920.
26. Abraham G, Kowalczyk A, Zobel J, Inouye M (2012) SparSNP: Fast and memory-efficient analysis of all SNPs for phenotype prediction. BMC Bioinformatics 13: 88.
27. Evans D, Visscher P, Wray N (2009) Harnessing the information contained within genome-wide association studies to improve individual prediction of complex disease risk. Human Molecular Genetics 18: 3525–3531.
28. Yang J, Lee SH, Goddard ME, Visscher PM (2011) GCTA: a tool for genome-wide complex trait analysis. American Journal of Human Genetics 88: 76–82.
29. Lee SH, Wray NR, Goddard ME, Visscher PM (2011) Estimating missing heritability for disease from genome-wide association studies. American Journal of Human Genetics 88: 294–305.
30. Abraham G, Kowalczyk A, Zobel J, Inouye M (2013) Performance and robustness of penalized and unpenalized methods for genetic prediction of complex human disease. Genetic Epidemiology 37: 184–195.
31. Wei Z, Wang W, Bradfield J, Li J, Cardinale C, et al. (2013) Large Sample Size, Wide Variant Spectrum, and Advanced Machine-Learning Technique Boost Risk Prediction for Inflammatory Bowel Disease. The American Journal of Human Genetics 92: 1008–1012.
32. Wray NR, Yang J, Goddard ME, Visscher PM (2010) The genetic interpretation of area under the ROC curve in genomic profiling. PLoS Genetics 6: e1000864.

33. Varma S, Simon R (2006) Bias in error estimation when using cross-validation for model selection. BMC Bioinformatics 7: 91.
34. Wood Ia, Visscher PM, Mengersen KL (2007) Classification based upon gene expression data: bias and precision of error rates. Bioinformatics 23: 1363–1370.
35. Zheng X, Shen J, Cox C, Wakefield JC, Ehm MG, et al. (2013) HIBAG – HLA Genotype Imputation with Attribute Bagging. Pharmacogenomics Journal [Epub ahead of print]
36. Monsuur AJ, de Bakker PI, Zhernakova A, Pinto D, Verduijn W, et al. (2008) Effective detection of human leukocyte antigen risk alleles in celiac disease using tag single nucleotide polymorphisms. PLoS ONE 3: e2270.
37. The Wellcome Trust Case Control Consortium (2007) Genome-wide association study of 14,000 cases of seven common diseases and 3,000 shared controls. Nature 447: 661–678.
38. Cotsapas C, Voight BF, Rossin E, Lage K, Neale BM, et al. (2011) Pervasive sharing of genetic effects in autoimmune disease. PLoS Genetics 7: e1002254.
39. Smyth DJ, Plagnol V, Walker NM, Cooper JD, Downes K, et al. (2008) Shared and distinct genetic variants in type 1 diabetes and celiac disease. The New England Journal of Medicine 359: 2767–2777.
40. Altman DG, Bland JM (1994) Diagnostic tests 2: predictive values. BMJ 309: 102.
41. Rubio-Tapia A, Van Dyke CT, Lahr BD, Zinsmeister AR, El-Youssef M, et al. (2008) Predictors of family risk for celiac disease: a population-based study. Clin Gastroenterol Hepatol 6: 983–987.
42. Bourgey M, Calcagno G, Tinto N, Gennarelli D, Margaritte-Jeannin P, et al. (2007) HLA related genetic risk for coeliac disease. Gut 56: 1054–1059.
43. Volta U, Tovoli F, Caio G (2011) Clinical and immunological features of celiac disease in patients with Type 1 diabetes mellitus. Expert Rev Gastroenterol Hepatol 5: 479–487.
44. Hin H, Bird G, Fisher P, Mahy N, Jewell D (1999) Coeliac disease in primary care: case finding study. BMJ 318: 164–167.
45. Harrell FEJ (2001) Regression Modeling Strategies: Springer.
46. Naiyer AJ, Hernandez L, Ciaccio EJ, Papadakis K, Manavalan JS, et al. (2009) Comparison of commercially available serologic kits for the detection of celiac disease. J Clin Gastroenterol 43: 225–232.
47. Reeves GE, Squance ML, Duggan AE, Murugasu RR, Wilson RJ, et al. (2006) Diagnostic accuracy of coeliac serological tests: a prospective study. Eur J Gastroenterol Hepatol 18: 493–501.
48. Lewis NR, Scott BB (2006) Systematic review: the use of serology to exclude or diagnose coeliac disease (a comparison of the endomysial and tissue transglutaminase antibody tests). Aliment Pharmacol Ther 24: 47–54.
49. Wong RC, Steele RH, Reeves GE, Wilson RJ, Pink A, et al. (2003) Antibody and genetic testing in coeliac disease.[see comment]. Pathology 35: 285–304.
50. Simell S, Hoppu S, Hekkala A, Simell T, Stahlberg MR, et al. (2007) Fate of five celiac disease-associated antibodies during normal diet in genetically at-risk children observed from birth in a natural history study. Am J Gastroenterol 102: 2026–2035.
51. Simell S, Kupila A, Hoppu S, Hekkala A, Simell T, et al. (2005) Natural history of transglutaminase autoantibodies and mucosal changes in children carrying HLA-conferred celiac disease susceptibility. Scand J Gastroenterol 40: 1182–1191.
52. Walker MM, Murray JA, Ronkainen J, Aro P, Storskrubb T, et al. (2010) Detection of celiac disease and lymphocytic enteropathy by parallel serology and histopathology in a population-based study. Gastroenterology 139: 112–119.
53. Biagi F, Klersy C, Balduzzi D, Corazza GR (2010) Are we not over-estimating the prevalence of coeliac disease in the general population? Ann Med 42: 557–561.
54. Maki M, Mustalahti K, Kokkonen J, Kulmala P, Haapalahti M, et al. (2003) Prevalence of Celiac disease among children in Finland. New England Journal of Medicine 348: 2517–2524.
55. Kurppa K, Ashorn M, Iltanen S, Koskinen LL, Saavalainen P, et al. (2010) Celiac disease without villous atrophy in children: a prospective study. J Pediatr 157: 373–380, 380 e371.
56. Kurppa K, Collin P, Viljamaa M, Haimila K, Saavalainen P, et al. (2009) Diagnosing mild enteropathy celiac disease: a randomized, controlled clinical study. Gastroenterology 136: 816–823.
57. Hopper AD, Hadjivassiliou M, Hurlstone DP, Lobo AJ, McAlindon ME, et al. (2008) What is the role of serologic testing in celiac disease? A prospective, biopsy-confirmed study with economic analysis. Clin Gastroenterol Hepatol 6: 314–320.
58. Hopper AD, Cross SS, Hurlstone DP, McAlindon ME, Lobo AJ, et al. (2007) Pre-endoscopy serological testing for coeliac disease: evaluation of a clinical decision tool. BMJ 334: 729.
59. NPD Group (January 2013) Dieting monitor: Eating Patterns in America. NPD Group.
60. Jones HJ, Warner JT (2010) NICE clinical guideline 86. Coeliac disease: recognition and assessment of coeliac disease. Arch Dis Child 95: 312–313.
61. Rubio-Tapia A, Hill ID, Kelly CP, Calderwood AH, Murray JA (2013) ACG clinical guidelines: diagnosis and management of celiac disease. Am J Gastroenterol 108: 656–676; quiz 677.
62. Rostom A, Murray JA, Kagnoff MF (2006) American Gastroenterological Association (AGA) Institute technical review on the diagnosis and management of celiac disease. Gastroenterology 131: 1981–2002.
63. Doolan A, Donaghue K, Fairchild J, Wong M, Williams AJ (2005) Use of HLA typing in diagnosing celiac disease in patients with type 1 diabetes. Diabetes Care 28: 806–809.
64. Matysiak-Budnik T, Malamut G, de Serre NP, Grosdidier E, Seguier S, et al. (2007) Long-term follow-up of 61 coeliac patients diagnosed in childhood: evolution toward latency is possible on a normal diet. Gut 56: 1379–1386.
65. Hadithi M, von Blomberg BM, Crusius JB, Bloemena E, Kostense PJ, et al. (2007) Accuracy of serologic tests and HLA-DQ typing for diagnosing celiac disease. Ann Intern Med 147: 294–302.
66. Trynka G, Hunt Ka, Bockett Na, Romanos J, Mistry V, et al. (2011) Dense genotyping identifies and localizes multiple common and rare variant association signals in celiac disease. Nature Genetics 43: 1193–1201.
67. Patterson N, Price AL, Reich D (2006) Population structure and eigenanalysis. PLoS Genet 2: e190.
68. Zhou X, Carbonetto P, Stephens M (2013) Polygenic Modeling with Bayesian Sparse Linear Mixed Models. PLoS Genetics 9: e1003264.
69. R Core Team (2012) R: A language and environment for statistical computing. Vienna, Austria: R Foundation for Statistical Computing.
70. Wickham H (2009) ggplot2: elegant graphics for data analysis. New York: Springer.
71. Hanley AJ, McNeil BJ (1982) The Meaning and Use of the Area under A Receiver Operating Characteristic (ROC) Curve. Radiology 143: 29–36.
